# Unsupervised clustering of longitudinal clinical measurements in electronic health records

**DOI:** 10.1371/journal.pdig.0000628

**Published:** 2024-10-15

**Authors:** Arshiya Mariam, Hamed Javidi, Emily C. Zabor, Ran Zhao, Tomas Radivoyevitch, Daniel M. Rotroff

**Affiliations:** 1 Department of Quantitative Health Sciences, Lerner Research Institute, Cleveland Clinic, Cleveland, Ohio, United States of America; 2 Center for Quantitative Metabolic Research, Cleveland Clinic, Cleveland, Ohio, United States of America; 3 Department of Electrical Engineering and Computer Science, Cleveland State University, Cleveland, Ohio, United States of America; 4 Taussig Cancer Institute, Cleveland Clinic, Cleveland, Ohio United States of America; 5 Endocrinology and Metabolism Institute, Cleveland Clinic, Cleveland, Ohio, United States of America; National Yang Ming Chiao Tung University, TAIWAN

## Abstract

Longitudinal electronic health records (EHR) can be utilized to identify patterns of disease development and progression in real-world settings. Unsupervised temporal matching algorithms are being repurposed to EHR from signal processing- and protein-sequence alignment tasks where they have shown immense promise for gaining insight into disease. The robustness of these algorithms for classifying EHR clinical data remains to be determined. Timeseries compiled from clinical measurements, such as blood pressure, have far more irregularity in sampling and missingness than the data for which these algorithms were developed, necessitating a systematic evaluation of these methods. We applied 30 state-of-the-art unsupervised machine learning algorithms to 6,912 systematically generated simulated clinical datasets across five parameters. These algorithms included eight temporal matching algorithms with fourteen partitional and eight fuzzy clustering methods. Nemenyi tests were used to determine differences in accuracy using the Adjusted Rand Index (ARI). Dynamic time warping and its lower-bound variants had the highest accuracies across all cohorts (median ARI>0.70). All 30 methods were better at discriminating classes with differences in magnitude compared to differences in trajectory shapes. Missingness impacted accuracies only when classes were different by trajectory shape. The method with the highest ARI was then used to cluster a large pediatric metabolic syndrome (MetS) cohort (N = 43,426). We identified three unique childhood BMI patterns with high average cluster consensus (>70%). The algorithm identified a cluster with consistently high BMI which had the greatest risk of MetS, consistent with prior literature (OR = 4.87, *95% CI*: 3.93–6.12). While these algorithms have been shown to have similar accuracies for regular timeseries, their accuracies in clinical applications vary substantially in discriminating differences in shape and especially with moderate to high missingness (>10%). This systematic assessment also shows that the most robust algorithms tested here can derive meaningful insights from longitudinal clinical data.

## Author summary

Clinical data is regularly recorded in patients’ health records by healthcare institutions and is becoming increasingly available for research to identify clinically meaningful subgroups, that can help drive developments in precision medicine. Clustering methods from other domains, such as audio signal processing, are being repurposed for these tasks however, clinical data has its own unique characteristics, such as missing data and specific correlation structures, that may impact the performance of certain clustering methods. Here, using a large, simulated dataset we developed from real patient data, our objective is to establish which approaches are best at stratifying patients using longitudinal clinical data. We identified dynamic time warping (DTW) and its lower-bound variants as highly robust clustering algorithms that showed impressive performance at classifying patients based on variations in trajectory shapes and trajectory magnitudes. We also demonstrate, using a real cohort of >43,000 pediatric patients, that DTW can classify BMI trajectories to identify patients at elevated risk of developing pediatric metabolic syndrome. Our study provides insights in the robustness of algorithms and their use in identifying novel pattens in clinical domain.

## Introduction

The application of unsupervised machine learning algorithms to longitudinal electronic health records (EHRs) offers unprecedented opportunities to identify patterns of clinical biomarkers that can improve health and derive new insights in disease progression from real world cohorts [1–6]. Historically, timeseries matching algorithms, such as dynamic time warping (DTW), have shown immense potential where timeseries intervals are regular, such as speech recognition, audio signal processing, and protein sequence alignments [7–10]. Timeseries matching algorithms can be used to measure similarity between patient’s longitudinal data. These similarity measures can then be used to identify clusters of patients with similarly trajectories, leading to new clinical insights [11,12]. However, the longitudinal data captured in the EHR differs substantially from the types of data traditionally used to evaluate these methods. For example, a patient’s clinical data is greatly influenced by many factors [5,6], resulting in non-random data missingness and irregular sampling that are not routinely seen in the other data types used to develop these methods. Systematic investigation of these algorithms using real-world clinical lab measurements is needed to determine which methods are the most accurate and robust for deriving clinically meaningful insights [13].

The challenge with using real-world data for comparing methods is that the true signal cannot be known, therefore we can never truly know what class a patient actually belongs to when clustered. The value of using simulated datasets is that we can identify and modulate the true signal, providing the ability to systematically compare clustering algorithms under different experimental conditions. However, simulated datasets need to be representative of real-world observations to make their findings generalizable. Here, we generated simulated datasets from real-world routine clinical measurements—body mass index (BMI), systolic blood pressure (SBP) and random glucose. This approach provides the advantage of deriving simulated cohorts with correlation structures and other key aspects that are observed in real EHR data, and enabled us to 1) assess the impact of various parameters on the robustness of clustering algorithms and 2) identify algorithms best suited for discovering clinical insights in EHR data[13].

Unsupervised clustering algorithms aim to find clusters based on similarities within the data, rather than supervised methods that try to learn clusters based on a set of pre-labeled observations. These unsupervised algorithms can be divided into centroid-based approaches and hierarchical approaches, both require a calculation of a similarity metric between the timeseries. Here, we focused on centroid-based approaches since hierarchical approaches require substantially more computational time and resources, limiting their ability to scale [14]. Unsupervised clustering algorithms can be separated into three major components: i) assignment method: patient is assigned to either one cluster (partitional clustering) or all clusters with varied probabilities (fuzzy clustering), ii) centroid computation: method to iteratively update the centroid of clusters and iii) distance metric: method to calculate distance between centroids and timeseries [14]. We evaluated different variations of assignment type, centroid computation and distance measures to form 30 unique clustering algorithms utilizing eight state-of-the-art timeseries matching algorithms as distance measures and apply these to simulated datasets to determine the most suitable algorithms for longitudinal EHR data.

**Fig 1:**
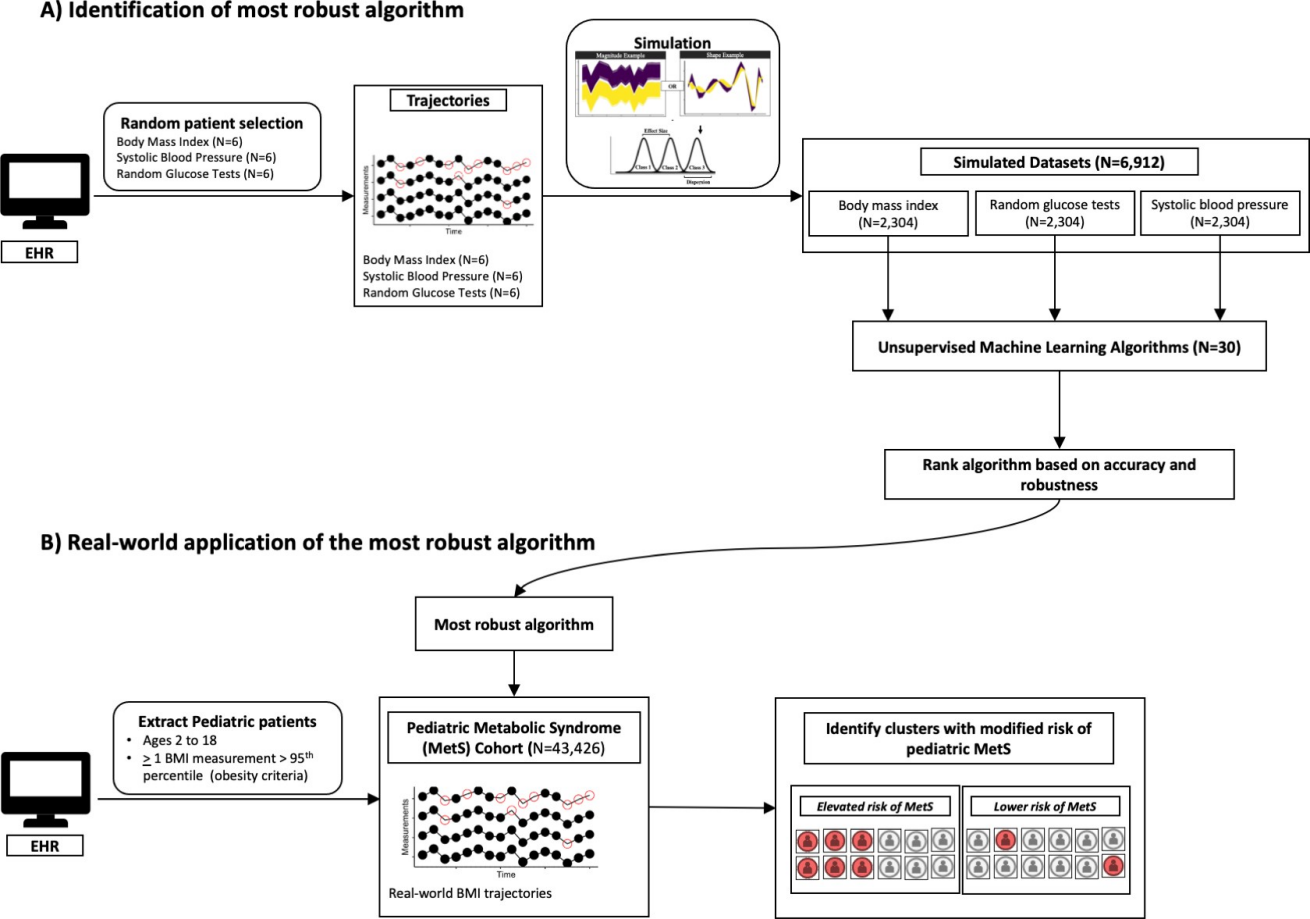
Study Design. Clinical data was randomly selected from patients’ electronic health record (EHR) data and used as basis for simulation. Unsupervised machine learning algorithms were applied to simulated datasets and their accuracies were ranked. The most suitable algorithm was then applied to a real-world pediatric cohort to identify BMI patterns with distinct risk of metabolic syndrome (MetS).

We then tested the utility of the most robust algorithm to examine childhood BMI patterns associated with risk profiles of metabolic syndrome (MetS) in a large real-world pediatric cohort (Fig 1). MetS is a combination of health conditions whose comorbidity has been linked to greater risks of atherosclerosis, coronary heart disease and stroke [15]. Obesity is a major driver of this condition, and with increases in childhood obesity, MetS warrants greater vigilance [16]. Prior research has already established that subjects with high BMI values have increased risk of being diagnosed with MetS, but how BMI trajectories contribute to this risk is not well understood [15]. Here, we explored the extent to which clustering using the most accurate and robust algorithm is congruent with earlier research and capable of uncovering novel childhood BMI patterns linked with MetS.

## Results

### Overall results

**Fig 2:**
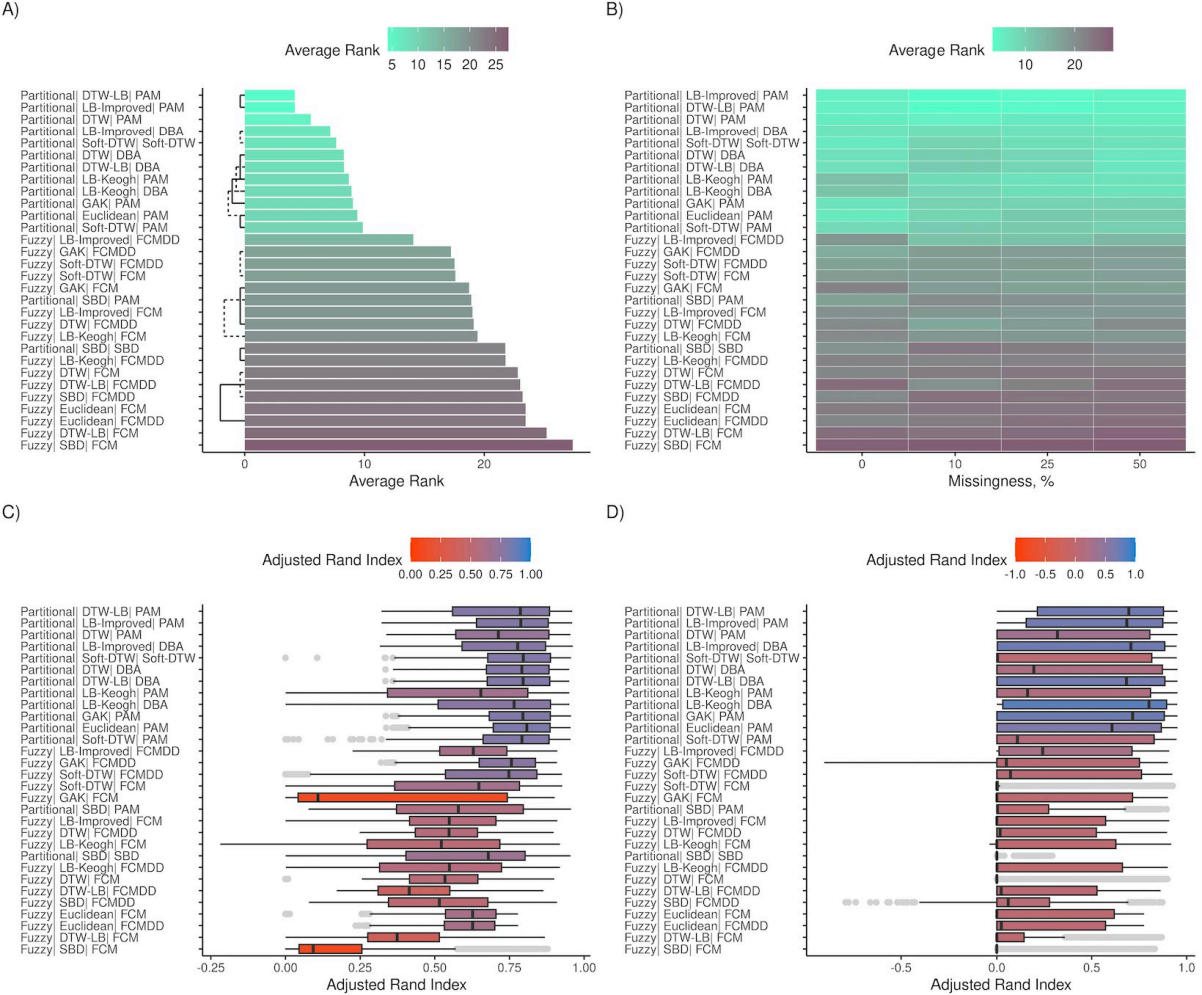
Algorithm ranking based on results from both magnitude and shape cohorts. (A-B) and (C-D) show the average rank and Adjusted Rand Index (ARI), respectively, for all 30 algorithms across all cohorts. Average ranks were obtained by comparing all algorithm using Nemenyi tests in the R package *mlr3benchmark*, and lower average ranks indicate better performance. ARI scale ranges between -1 to 1, and values closer to zero represent classification on par with random assignment. (A) and (B) shows average ranks. Algorithms with similar accuracies are connected by black bars (dashed & solid) in (A). These metrics are further subset by missingness in (B), (C) and (D). (C) and (D) show ARI distributions for simulated datasets with no missingness and missingness > 10%.

The 30 clustering algorithms were formed by combining two clustering assignment methods, six centroid computation methods and eight distance measures. Each method is described in detail in the supplemental information (S1 Text). The accuracy of clustering algorithms was assessed by calculating the Adjusted Rand Index (ARI) which ranges from -1 to 1 with values closer to zero indicating random sorting. The majority of the algorithms had median ARI closer to 1 indicating better cluster assignment than random chance (Fig 2).

Across all simulated datasets, algorithms with partitional clustering ranked higher than fuzzy clustering (Fig 2A). Methods for computing centroids included those specifically developed for timeseries (e.g., DTW Barycenter Averaging (DBA)) and generalized methods used for many data types (e.g., partitioning around medoids (PAM)). Surprisingly, PAM was used in three of the top five ranked algorithms, outperforming methods developed specifically for timeseries.

DTW-lower bounding (LB) and LB-Improved distance metric tied for the first rank (mean ranks = 4.19, *P* > .05) (Fig 2A–2B). DTW ranked third (mean rank = 5.53, *Ps* < .05). The top three ranks belonged to the same three algorithms, i.e., DTW-LB, LB-Improved and DTW with PAM centroids, when datasets were subset by clinical measurement types (i.e., BMI, random glucose, SBP) (S8, S15 and S22 Figs).

**Fig 3:**
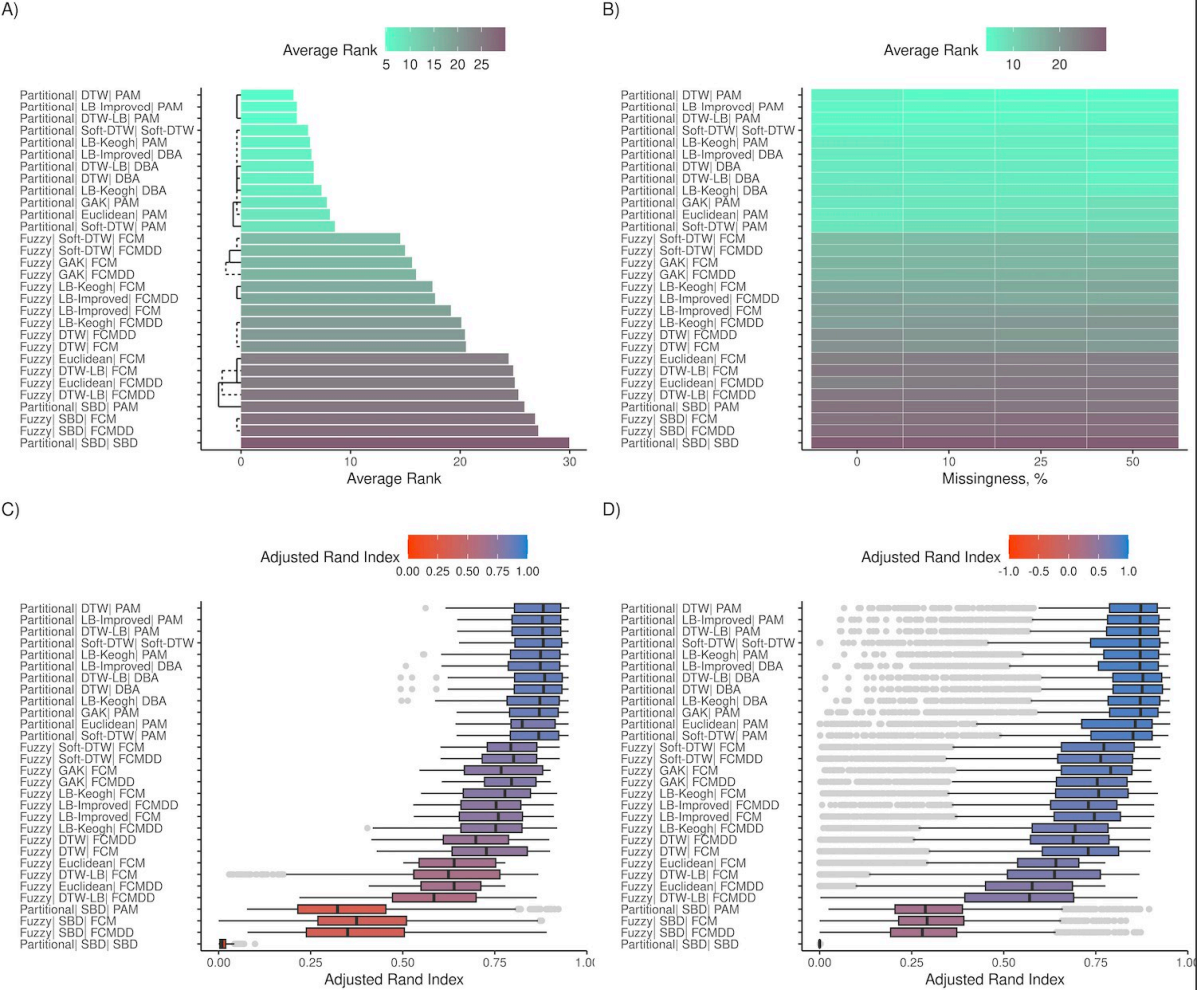
Algorithm rankings for magnitude cohorts. (A-B) and (C-D) show the average rank and Adjusted Rand Index (ARI), respectively, for all 30 algorithms across magnitude cohorts. Average ranks were obtained by comparing all algorithm using Nemenyi tests in the R package *mlr3benchmark*, and lower average ranks indicate better performance. ARI scale ranges between -1 to 1, and values closer to zero represent classification on par with random assignment. (A) and (B) shows average ranks. Algorithms with similar accuracies are connected by black bars (dashed & solid) in (A). These metrics are further subset by missingness in (B), (C) and (D). (C) and (D) show ARI distributions for simulated datasets with no missingness and missingness > 10%.

**Fig 4:**
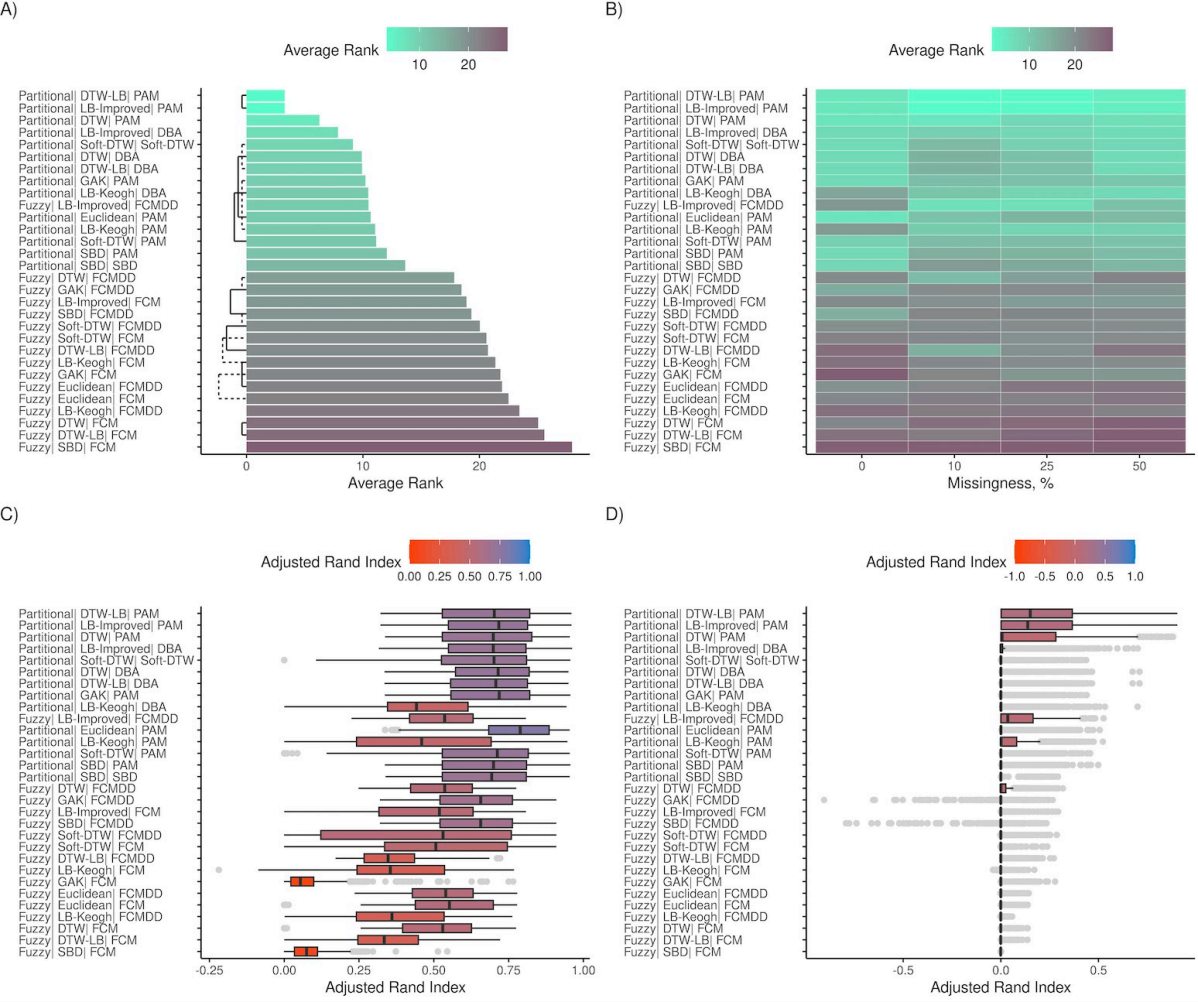
Algorithm rankings for shape cohorts. (A-B) and (C-D) show the average rank and Adjusted Rand Index (ARI), respectively, for all 30 algorithms across shape cohorts. Average ranks were obtained by comparing all algorithm using Nemenyi tests in the R package *mlr3benchmark*, and lower average ranks indicate better performance. ARI scale ranges between -1 to 1, and values closer to zero represent classification on par with random assignment. (A) and (B) shows average ranks. Algorithms with similar accuracies are connected by black bars (dashed & solid) in (A). These metrics are further subset by missingness in (B), (C) and (D). (C) and (D) show ARI distributions for simulated datasets with no missingness and missingness > 10%.

**Fig 5:**
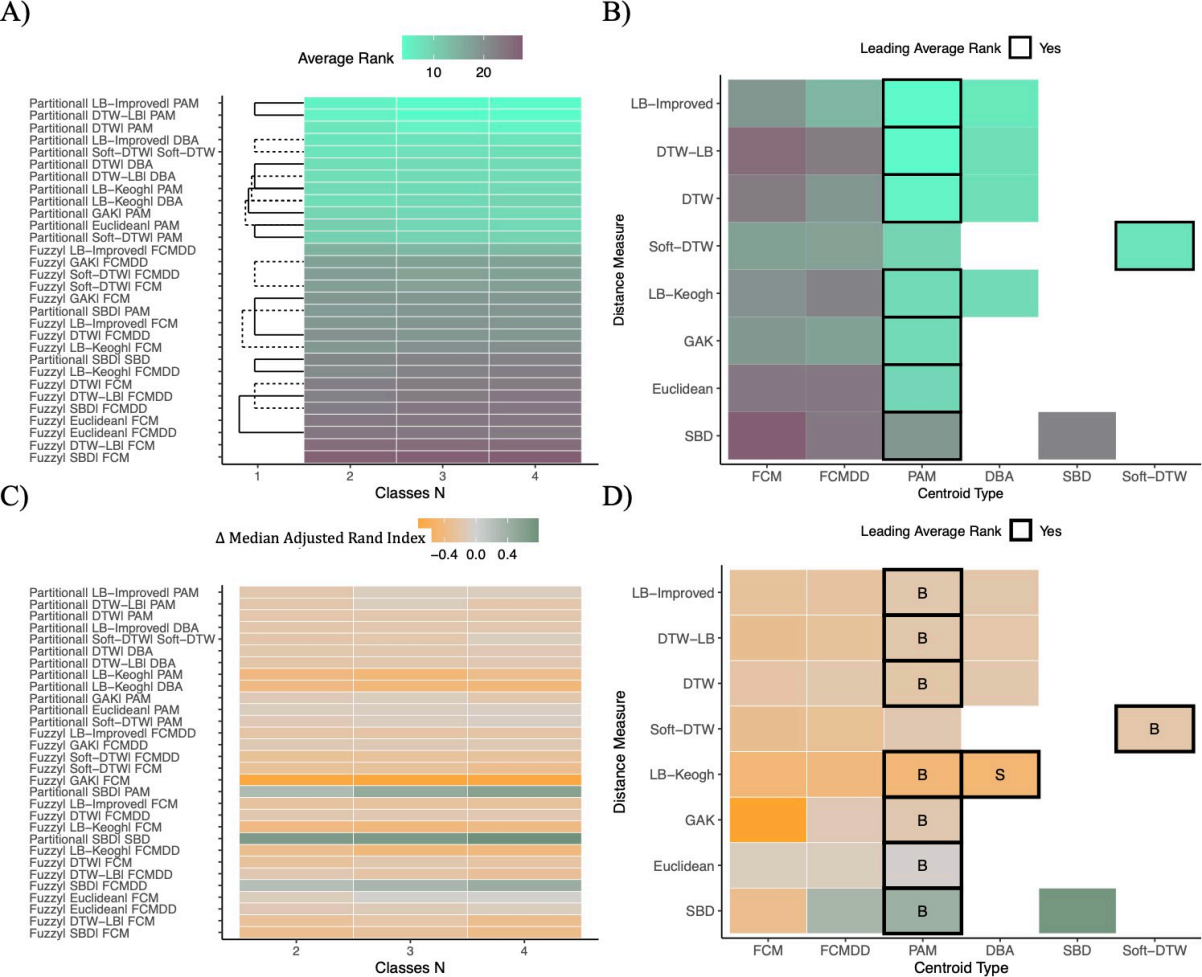
Impact of cohort type and number of classes on algorithms. Algorithm accuracies were compared using Nemenyi tests in R package *mlr3benchmark*. Algorithms with similar accuracies are shown by the black bars in (A) and (C). (A) Average rank of the algorithms across all datasets is shown by the number of classes. (B) Average ranks for combinations of centroids and distance measures used. (C-D) Difference in median ARI between magnitude and shape cohorts. ΔARI>0 indicates higher median accuracies in shape cohorts than magnitude cohorts. ΔARI is stratified by number of classes in (C). (D) is annotated with “M” if the combination ranked significantly higher in magnitude cohorts, “S” if it ranked significantly higher in shape cohorts and “B” if rankings did not differ between magnitude and shape cohorts.

As expected, accuracies decreased as missingness increased in the simulated cohorts (Fig 2B). Furthermore, as the overlap between classes increased, controlled by dispersion and effect sizes, ARI for all algorithms showed a downward trend (Fig 2E). All five algorithms were better at finding true patterns in cohorts with trajectory magnitude differences than cohorts with trajectory shape differences (Figs 3 and 4). Methods were ranked consistent when simulated cohorts had 2, 3 and 4 true classes (Fig 5A). Nearly, all distance measures ranked higher with PAM centroids than with other centroids, with the only exceptions being Soft-DTW, which was most robust when used with Soft-DTW centroids (Fig 5B).

### Magnitude simulation results

While differences in accuracies of top five algorithms were observed in the overall results, the top five algorithms in magnitude cohorts were equally accurate (Mean ranks = 4.78–5.10) (*Ps* > .05) (Fig 3). In order of mean ranks, DTW, LB-Improved and DTW-LB distance measures with PAM centroids were the three most accurate methods. LB-Keogh was considerably more robust in magnitude cohorts than in shape cohorts (ΔARI < -0.4) (Fig 5C). The subsequent fourth and fifth ranks were Soft-DTW (Mean rank = 6.13) and LB-Keogh (Mean rank = 6.32), latter with PAM centroids. It is noteworthy that the performance of these two algorithms varied such that they also tied with LB-Improved with DBA centroids (Mean rank = 6.43) (*Ps* > .05). Increasing missingness did not impact mean accuracies of any of these algorithms (Fig 3B).

### Shape simulation results

Robustness of algorithms varied greatly in shape cohorts (Fig 4A–4D). Overall, algorithms had lower accuracies in the shape cohorts than in the magnitude cohorts across every parameter, indicating that clustering based on differences in shape is more challenging than clustering based on differences in magnitude (Fig 4C). Missingness also had a greater impact on the accuracies in differentiating shapes compared to magnitudes (Fig 4B and 4D), with no algorithms performing well on shape cohorts with >10% missingness.

DTW-LB and LB-Improved (Mean ranks = 3.29) distances with PAM centroids had the greatest mean accuracies for the shape cohorts (Fig 4A–4D). DTW with PAM centroids was a close third (Mean rank = 6.26, *Ps*<0.05). The same three timeseries matching algorithms occupied three of next four ranks with DBA centroids. Shape-based DTW (SBD) was the only algorithm with greater accuracies in shape cohorts compared to magnitude cohorts (Fig 5D), however its overall ranking was low.

### Clustering of real-world pediatric BMI trajectories from the MetS cohort

After preprocessing, the cohort consisted of 43,426 children between the ages of 2 and 18 who were seen at Cleveland Clinic between 01/01/2000 and 12/31/2020 with at least one BMI exceeding 95^th^ percentile, the age-based criteria for obesity. The cohort was predominantly male (55.7%) and Caucasian (73.9%) (Table 1). Approximately 3.4% (N = 1,474) of the cohort either met the criteria for MetS or was diagnosed with MetS. Mean follow-up duration for the cohort was 8.47 years (SD = 3.60), and was significantly different between children with MetS and children without MetS (7.17 vs. 8.52, *P* < .001) (S3 Table). However, these differences were limited to the final follow-up age suggesting that children with and without MetS were followed consistently through young ages (*P* = .81) (S3 Table). Encounters with weight measurements were observed for 47.7% and 41.5% of children without and with MetS after World Health Organization declared coronavirus disease 2019 (COVID-19) a public health emergency of international concern. However, the percentage of MetS cases diagnosed in 2019 and 2020 were not significantly different (4.2% vs. 3.6%, *P* = 0.61).

**Table 1:** Descriptive statistics for pediatric metabolic syndrome cohort.

N	43,426
**Gender**, Male (%)	24,195 (55.7)
**Race** (%)	
American Indian or Alaska Native Asians Black race Caucasian Multiracial Unknown	38 (0.1) 533 (1.2) 6,564 (15.1) 32,081 (73.9) 2,172 (5.0) 2,038 (4.7)
**First age** (mean (SD))	4.99 (3.60)
**Last age** (mean (SD))	13.46 (3.72)
Follow-up time, years (mean (SD))	8.47 (3.54)
Metabolic Syndrome (%)	1,474 (3.4)

**Fig 6:**
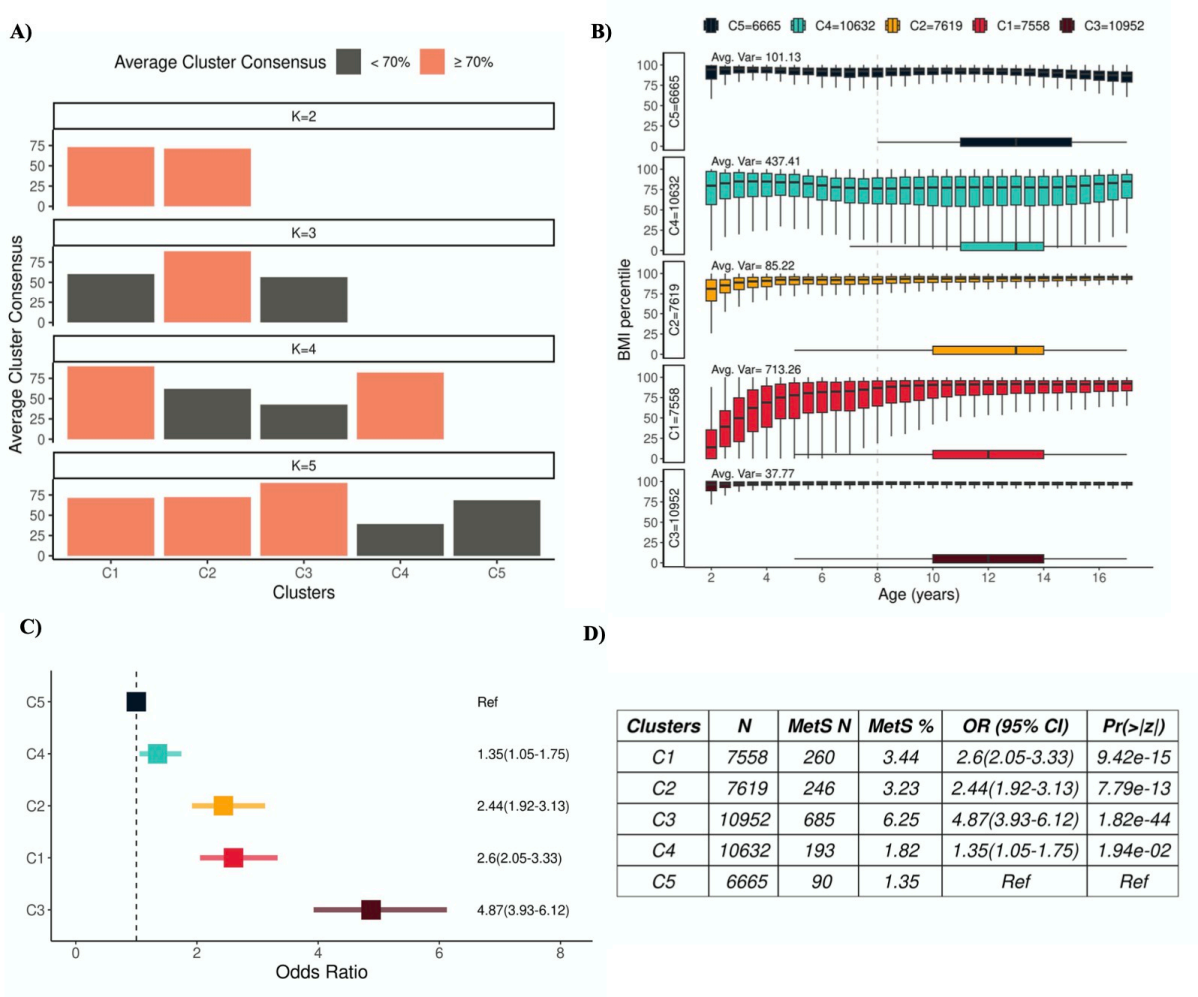
MetS Clusters. (A) Average cluster consensus for different numbers of clusters. (B) Body-mass-index (BMI) percentile distributions by age for each cluster. Horizontal boxplots show age distributions of MetS diagnoses in clusters. (C) Odds ratio for each cluster obtained using logistic regression with C5 as the reference. (D) Results from logistic regression models for association with MetS.

Partitional clustering with DTW as the distance measure and PAM centroids was applied to the pediatric BMI trajectories because i) along with LB-Improved, it was one of the three most accurate algorithms in the overall tests, magnitude tests and shape tests and ii) unlike LB-Improved, it is robust to variance in the length of trajectories. Since the true number of underlying clusters in the cohort were unknown, we utilized internal validation metrics to determine the optimal number of clusters (see Methods for details). Clustering identified by at least one of three internal validation metrics as optimal and with average pairwise consensus greater than 70% was selected for subsequent risk analysis. Dunn’s index identified five as the optimal number of clusters (k). At k = 5, the average resampling consensus of the three clusters (i.e., C1-C3) was >70% (Fig 6A). C4 and C5 had low average consensus suggesting lack of a systematic pattern driving cluster formation.

Children in C5 maintained a more stable BMI over the age range, with a slight downward trajectory at older age ranges (variance = 101.13). In contrast, children in C3 had consistently higher BMI than children in other clusters and this cluster also had the greatest risk of MetS (OR = 4.87, *95% CI*: 3.93–6.12 compared to C5), consistent with prior literature [17,18]. Children whose BMI increased with age, as seen in C1 and C2, were also at elevated risk of MetS compared to children with stable lower BMIs in C5 (Fig 5B–5D). Compared to C5, C1 and C2 had odds ratios for developing MetS of 2.44 (*95% CI*: 1.92–3.13) and 2.60 (*95% CI*: 2.05–3.33), respectively. The risk of MetS was also elevated in C4 compared to C5 (OR = 1.35, *95% CI*: 1.05–1.75), and C4 demonstrated much greater BMI variation (variance = 437.41) (Fig 6B).

## Discussion

Unsupervised machine learning algorithms have the potential to derive new insights in disease development, progression, and response to treatment [11,12]. Established timeseries matching algorithms have shown promise in fields such as signal processing and interest in their application in clinical data is growing. However, the accuracies and robustness of these algorithms in clinical data has not been systematically studied. Hence, our aim was to leverage simulated datasets developed from a several common clinical measurements (i.e., BMI, SBP, random glucose) to evaluate 30 unsupervised clustering algorithms composed, in part, of eight state-of-the-art timeseries matching algorithms. DTW is well-established as the gold standard for timeseries classification [19,20]. Our findings support that DTW and its variants (i.e., LB-Improved and DTW-LB) can more accurately identify underlying longitudinal patterns in clinical measurements than the other methods evaluated here. The top three ranks were occupied by distance measures with PAM centroids in all simulated cohorts. Although DBA was originally devised to calculate representative centroids with DTW [21], distance measures with DBA centroids did not outperform the same distance measures with PAM centroids (Fig 4B). Partitioning methods ranked higher than fuzzy methods for all distance measures and centroid types. We used ARI for hard partitioning clustering and soft ARI for fuzzy clustering. While both ARI methods are identical in extreme cases of perfect classification and random assignment, soft ARI tends to be lower as overlap between classes increases [22,23]. However, algorithm rankings changed within fuzzy clustering suggesting that the centroid type impacted algorithm performance as well. While we did not assess the contribution of centroid types in assignment and degree of overlap on the soft ARI estimate, their impact should be considered in future applications of these algorithms.

All algorithms were more accurate in finding clusters with distinct patterns of magnitude than clusters with distinct patterns of shape. Notably, using PAM and DBA centroids in shape cohorts, LB-Improved, DTW and DTW-LB were the only algorithms with high accuracies (Fig 4A). DTW-LB leveraged LB-Improved for an initial estimate of similarity between timeseries which is followed by DTW to calculate similarities between timeseries [24]. LB-Keogh was the only algorithm with similar accuracies for both centroids in the shape cohorts (Fig 4D). LB-Improved with DBA centroids was moderately to highly accurate in tests of both the magnitude and shape datasets (Fig 2). DTW-LB and LB-Improved emerged as two of the most accurate classification algorithms for classes differing in shape. However, their ability to derive novel insights from the EHR may be limited, since these algorithms cannot accommodate trajectories with varied lengths [24].

Non-random missing data due to varying health care utilization and social determinants are common challenges for longitudinal data analyses in EHR [5,6]. Our findings suggest that missingness as low as 10% greatly reduces the accuracies of these methods in clinical data where differences in shape were the only difference between classes, and future studies using these methods in an unsupervised framework should prioritize complete data with narrow magnitude ranges. However, in cases aiming to predict classes, supervised deep learning models can be used as these have been found to be robust to data missingness as high as 50% [13]. In addition to partitional and fuzzy clustering methods, hierarchical clustering methods are often used in conjunction with temporal matching algorithms [21]. Studies have reported DTW with agglomerative hierarchical clustering to be highly accurate [12,25]. Hierarchical clustering methods are computationally intensive because they require complete pairwise comparisons of the trajectories. The utility of these algorithms relative to partitional and fuzzy methods remains to be investigated.

MetS causes substantial decreases in quality of life and increases in healthcare costs, and its incidence has been increasing with the rising incidence of obesity in children [15]. Here, we focus on identification of childhood BMI patterns with high risk of developing MetS. Since BMI measurements are regularly recorded in clinical practice, these patterns may be useful to inform the need for weight management interventions. We identified three algorithms suitable for application in clinical data. DTW with PAM centroids repeatedly emerged as one of the three most accurate algorithms and was applied to pediatric MetS cohort based on its ability to handle non-uniform trajectory lengths. It optimized at five clusters, with three of these clusters representing distinct BMI trajectories (average cluster consensus >70%) (Fig 5A). Consistent with previous work [18], we also found elevated risk of MetS in the cluster with consistently high BMI (C3) (Fig 6B–6D). While we did not observe differences in ages of the start of trajectories between children with MetS and without MetS (S3 Table), the mean age for the start of BMI trajectories in C3 was later (Mean = 6.29 years, SD = 3.90) suggesting access to healthcare may have started later for this cluster than others. Prevalence estimates of MetS in females tends to be higher than in males [18]. C3 also had highest proportion of females (N = 5,479, 50%) compared to other clusters (S4 Table). While C3 also had a greater percentage of non-White individuals, this cluster was not overrepresented for non-White MetS cases (*P* = 0.06) (S5 Table). C4 and C5 had similarly lower BMI profiles; however, C4 had higher risk of MetS (OR = 1.35, *95% CI*: 1.05–1.75). This may be due to higher BMI variability in C4 as evident in the cluster’s low average consensus and high mean variance (Fig 6). All clusters, except C5, had cases of MetS diagnosed by physicians before 8 years of age (Fig 6B). Lowest and later MetS risk was associated with the C5 cluster with stable lower BMI percentiles over time. Studying MetS is challenging due to the relative ambiguity of how MetS is diagnosed. Instead of relying solely on ICD codes, we also incorporated lab measurements to reduce bias due to underdiagnoses. However, there may still be undiagnosed cases in our cohort since lab measurements may be ordered once MetS or another underlying condition is already suspected [15]. Pediatric BMI trajectories were included if BMI measurements were recorded annually in the EHR, but this introduces a potential for selection bias by enriching the cohort with children who had an overall greater health burden than the average pediatric population. Our investigation into BMI patterns with modified risk of MetS leverages longitudinal data only. These patterns were enriched for both MetS and certain demographic variables (e.g., sex) suggesting that interactions between BMI history and demographics provide valuable information important for studying the development of MetS. Future research should take demographic and prior medical history into account for more comprehensive risk profiling. Prior research has used this information in development of models of MetS in adults. These models used genetic and/or clinical information to accurately discriminate 65% to 93% of the individuals [26,27]. Important variables in these models included markers of dyslipidemia and hypertension prior to MetS diagnoses. However, it is important to note that these indicators are more common in adult medical histories compared to pediatric medical histories [15]. Here, our real-world application is important to show that longitudinal patterns of BMI are important indicators of MetS and in our affiliated deep learning work, we show that incorporation of these longitudinal features in prediction models greatly improve model accuracies [13,28].

We randomly selected six trajectories per clinical measurement type as basis for systematic manipulation of signal and noise in our simulation workflow. This enabled us to compare algorithm performance in a variety of scenarios where the underlying EHR-specific correlation structure is preserved and ground truth is known however, it is important to note that these trajectories do not represent all correlation structures observed in EHR and algorithm performances may vary under other circumstances such as different clinical lab measurements. However, it is important to note that the top algorithm performance ranks remained consistent across clinical measurement types in our analyses (i.e., BMI, random glucose & SBP) (Figs 2, S8, S15 and S22). Many of the algorithms (e.g., DTW-LB) also required the same time intervals for all trajectories and we used one of the simpler imputation methods, i.e., mean, to accommodate this requirement in our simulation analyses. More sophisticated imputation methods may improve the power of these algorithms in real world trajectories. While we accounted for variation due to random initialization of centroids by fitting these algorithms with five random seeds, we did not investigate the impact of initial conditions of the accuracies of the algorithms. The impact of the interaction between initial conditions and class overlap should be the evaluated in future studies. We accounted for initial conditions by taking consensus of the partitions in the pediatric MetS cohort. Our simulations included three common and representative clinical measurements (BMI, glucose, SBP), but these methods may perform differently on other clinical measurements.

We systematically simulated datasets across multiple parameters to characterize how well 30 unsupervised clustering algorithm identify clusters. We then applied one of the most robust algorithms identified to a real-world dataset to generate new insights into an increasingly important pediatric disorder. As true class membership is not known in real-world data, utilizing methods that demonstrate robust classification accuracies in similar simulated datasets provides additional confidence for finding cryptic substructure in clinical cohorts.

## Methods

### Data

Data from patients seen at Cleveland Clinic between 2000 and 2020 were extracted from the EHR and used to generate the simulated datasets and to extract the pediatric MetS cohort (Fig 1). We compiled timeseries from BMI, SBP and random glucose testing and randomly selected timeseries from six patients per measurement type to generate simulated datasets (Fig 1). These three different types of measurements were used as the basis for simulations to ensure representation of the varied longitudinal patterns observed in real-world clinical settings. Only BMI timeseries were compiled for the pediatric MetS cohort. Details for simulated datasets and MetS cohort are presented below.

### Simulated datasets

**Fig 7:**
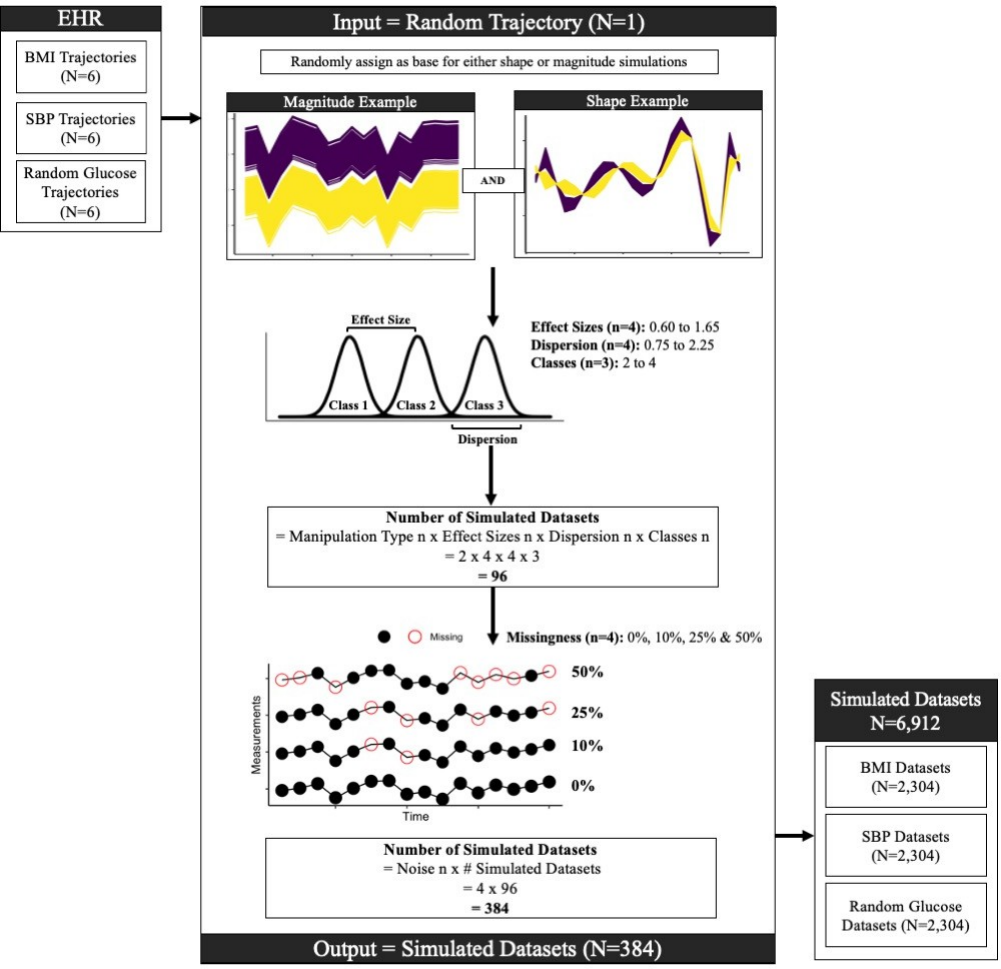
Simulation Workflow. Six trajectories were randomly selected for each type of clinical measurements i.e., body-mass-index (BMI), systolic blood pressure (SBP) and random glucose tests. The center panel shows the generation of simulated datasets for one real trajectory. Simulated trajectories were derived from the original trajectory by modifying its shape and magnitude separately. Effect size modulated the difference between the mean of the original and simulated class distributions. Effect size, together with dispersion around mean, modulated the overlap between these classes. Number of classes and data missingness were also simulated. This resulted in 384 simulated datasets per randomly selected trajectory.

We randomly selected six trajectories for each type of clinical lab type as basis for shape cohorts and magnitude simulations (Fig 7). These clinical lab types were selected based on their routine use for screening cardiometabolic conditions such as hypertension and diabetes [15]. BMI was calculated from routine weight and height measurements captured during encounters. Systolic blood pressure measurements were also captured during encounters. LOINC code 2339–0 was used to retrieve random glucose level measured in whole blood. Missing measurements were imputed using the mean values immediately before and after the missing value resulting in each trajectory with yearly measurements spanning 16 years. This ensured that the impact of missingness was explicitly manipulated in the simulation, as described below. As previously described in Javidi et al [13], differences between the classes were based on either magnitude, while holding trajectory shape constant (magnitude cohorts) or shape, while holding magnitude constant (shape cohorts). Changes in magnitude were introduced by randomly sampling from normal distributions of magnitudes. Changes in shape were introduced by randomly sampling from normal distributions of what was determined to be the most important shape parameter. The most important shape parameter was determined by fitting a polynomial regression to each trajectory. Regression parameters were then substituted with permutated values to determine the standard error between the trajectory and the polynomial regression. The most important parameter for influencing shape was determined to be the one with the greatest standard errors upon permutation. Code to identify the most important shape parameter has been made available: https://github.com/rotroff-lab/unsupervised_EHR_clustering.

We also varied the number of true classes in the dataset (n = 3), effect sizes based on normal distributions with prespecified means (n = 4), and dispersions based on standard deviations (n = 4), which also led to differences in the proportion of overlap between the classes (Fig 7). Overlap was higher for classes with greater dispersion and smaller effect sizes. The overlap was calculated as the number of trajectories belonging to multiple classes divided by total number of trajectories in the dataset and was assessed as a separate parameter. Effect sizes ranged from 0.60 to 1.65, and the dispersions were 0.75, 1.25, 1.75, and 2.25. The effect of missingness on ARI was evaluated by simulating data with 0%, 10%, 25% and 50% missingness. The combination of all varied parameters resulted in 384 datasets per patient, or 2,304 datasets per measurement type (i.e., BMI, SBP and glucose). Total number of simulated datasets was 6,912.

### Pediatric metabolic syndrome (MetS) dataset

The real-world cohort consisted of pediatric patients (ages 2–18 years old) seen at Cleveland Clinic between 01/01/2000 and 12/31/2020 with at least one BMI >95^th^ percentile, representing obesity. BMI trajectories were utilized for the real-world analyses as obesity is a major known driver of MetS and BMI, a marker of obesity, is routinely captured in EHR for children. Children who had at least one weight and/or height measurement per year of age were included and BMI percentiles were calculated and preprocessed using the R package *growthcleanr* [29]. Curated BMI measurements were then compiled from ages 2 up to 18 for patients without MetS and from ages 2 up to the age at diagnosis for patients with MetS. Splines were fit to smooth BMI trajectories and obtain BMI percentile annotations for each half year of age. Prior to smoothing, the median number of distinct encounters with weight measurements in the cohort were 30 (Interquartile range = 18–47). Based on standard guidelines, children (age >8 years) with at least three of the following risk factors have MetS: i) obesity, ii) elevated blood pressure, iii) low HDL-C, iv) high triglycerides levels and v) poor glycemic control [15,30]. In this study, cases had MetS if i) the physician diagnosed the patient as having either ICD-9 code 277.7 or ICD-10 code E88.81 regardless of age or ii) three of five conditions were met in a health record age eight onwards. The measurement variables and criteria for these conditions are listed in S1 Table. This study was approved by Cleveland Clinic Institutional Review Board (IRB # 20–135).

### Unsupervised machine learning

Centroid-based methods include both partitional and fuzzy clustering approaches. Partitional clustering assigns a trajectory to a specific cluster, while fuzzy clustering produces probabilities of the trajectory belonging to all clusters [14]. A variety of methods are used to compute centroids (e.g., PAM, DBA) and distances of timeseries from the centroids (e.g., DTW, DTW-LB). These methods are defined in S2 Table. In total, 29 clustering methods were applied to each simulated dataset by combining two partitioning types, eight distance measures and six centroid computation types. If a centroid computation method was developed for a specific distance measure, it was only applied in conjunction with that distance measure. The R package dtwclust was used to implement these methods [24]. Additional details are provided in Supplementary Methods (S1 Text).

### Identification of most robust algorithm based on ranking of clustering approaches

Accuracies of the algorithms were evaluated by calculating Adjusted Rand Index (ARI) for partitional clustering and soft ARI for fuzzy clustering. Both ARI and soft ARI range from -1 to 1 with values closer to zero indicating that the algorithm is on par with random assignment. ARI and soft ARI tend to be identical in cases of perfect classification and random assignment [22]. The distributions of ARI across simulated datasets were compared for each pair of algorithms using Nemenyi tests and performances were ranked. Nemenyi test [31] assumes a null hypothesis of no difference between the accuracies of the compared approaches. Approaches were compared across all simulated datasets as well as separately in the magnitude and shape simulation datasets. Benjamini Hochberg method was used to adjust *P* values for multiple comparisons [32]. FDR-adjusted *P* values < .05 indicated significant differences. Tests were performed, and an average ranking based on critical difference was computed, using the R package *mlr3benchmark* [33]. The algorithm with consistently greater ARI was then applied to the real-world cohort of pediatric BMI trajectories to identify patterns associated with increased risk of MetS.

### Real-world application in pediatric MetS dataset

The most robust algorithm identified in the simulated datasets was applied to the pediatric MetS dataset. Unlike the simulated datasets, the classes in the real-world cohort were not known and since ARI requires knowledge of true classes, it was replaced with internal validation metrics for real-world application. The dataset was clustered with five random seeds. The optimal number of clusters (k) was identified using the following internal validation metrics: Dunn’s index, Ibai Gurrutxaga (COP) index and Silhouette Index. Internal validation metrics assess clustering partitions based on clustered data only with the objective of finalizing compact clusters while maximizing differences between them. Consensus-based measures (i.e., average cluster consensus) were calculated to assess the stability of the clusters [34]. At each k, the average number of times two individuals clustered together across five random seeds was also calculated and K-modes was applied for final clustering assignments. We selected the clustering with greatest number of clusters supported by at least one internal validation metric, and with average pairwise consensus greater than 70%. Enrichment of MetS in each cluster was investigated using logistic regression.

## Supporting information

S1 Text Supplementary Methods. (DOCX)

S1 Table Metabolic Syndrome Criteria. (DOCX)

S2 Table Algorithms applied to simulated datasets. (DOCX)

S3 Table Descriptive statistics by MetS status. (DOCX)

S4 Table Descriptive statistics of clusters found in MetS cohort. (DOCX)

S5 Table Descriptive statistics of clusters in MetS cases only. (DOCX)

S1 Fig Adjusted Rand Index Distributions for algorithms for all cohorts. (DOCX)

S2 Fig Algorithm ranking for all magnitude cohorts. (A) shows the average rank of the algorithms. Algorithm rankings were compared using the Nemenyi tests in R mlr3benchmark package. Algorithms with similar accuracies are shown by the black bars in (A). There are no differences between dashed and solid bars. (B) shows the average rank of the algorithms by missingness levels. (C) shows the ARI distribution for cohorts with no missingness (D) shows the ARI distribution for cohorts with missingness.
(DOCX)

S3 Fig Impact of cohort type and number of classes on algorithms for all magnitude cohorts. Algorithm accuracies were compared using the Nemenyi tests in R mlr3benchmark package. Algorithms with similar accuracies are shown by the black bars in (A).
(DOCX)

S4 Fig Adjusted Rand Index Distributions for algorithms for all magnitude cohorts. (DOCX)

S5 Fig Algorithm ranking for all shape cohorts. (A) shows the average rank of the algorithms. Algorithm rankings were compared using the Nemenyi tests in R mlr3benchmark package. Algorithms with similar accuracies are shown by the black bars in (A). There are no differences between dashed and solid bars. (B) shows the average rank of the algorithms by missingness levels. (C) shows the ARI distribution for cohorts with no missingness (D) shows the ARI distribution for cohorts with missingness.
(DOCX)

S6 Fig Impact of cohort type and number of classes on algorithms for all shape cohorts. Algorithm accuracies were compared using the Nemenyi tests in R mlr3benchmark package. Algorithms with similar accuracies are shown by the black bars in (A).
(DOCX)

S7 Fig Adjusted Rand Index Distributions for algorithms for all shape cohorts. (DOCX)

S8 Fig Algorithm ranking for BMI. (A) shows the average rank of the algorithms. Algorithm rankings were compared using the Nemenyi tests in R mlr3benchmark package. Algorithms with similar accuracies are shown by the black bars in (A). There are no differences between dashed and solid bars. (B) shows the average rank of the algorithms by missingness levels. (C) shows the ARI distribution for cohorts with no missingness (D) shows the ARI distribution for cohorts with missingness.
(DOCX)

S9 Fig Impact of cohort type and number of classes on algorithms for BMI. Algorithm accuracies were compared using the Nemenyi tests in R mlr3benchmark package. Algorithms with similar accuracies are shown by the black bars in (A).
(DOCX)

S10 Fig Difference in algorithm accuracies by cohort type and number of classes for BMI. Algorithm accuracies were compared using the Nemenyi tests in R mlr3benchmark package. Algorithms with similar accuracies are shown by the black bars in (A). (A) and (B) show the difference in median ARI between magnitude and shape cohorts. ΔARI>0 indicates higher median accuracies in shape cohorts than magnitude cohorts. ΔARI is stratified by number of classes in (A). (B) shows ΔARI for combinations of centroids and distance measures used. The annotations B, M, and S refer to the algorithm combination ranking higher in both, magnitude only and shape only cohorts, respectively.
(DOCX)

S11 Fig Algorithm ranking for BMI magnitude cohorts. (A) shows the average rank of the algorithms. Algorithm rankings were compared using the Nemenyi tests in R mlr3benchmark package. Algorithms with similar accuracies are shown by the black bars in (A). There are no differences between dashed and solid bars. (B) shows the average rank of the algorithms by missingness levels. (C) shows the ARI distribution for cohorts with no missingness (D) shows the ARI distribution for cohorts with missingness.
(DOCX)

S12 Fig Impact of cohort type and number of classes on algorithms for BMI magnitude cohorts. Algorithm accuracies were compared using the Nemenyi tests in R mlr3benchmark package. Algorithms with similar accuracies are shown by the black bars in (A).
(DOCX)

S13 Fig Algorithm ranking for BMI shape cohorts. (A) shows the average rank of the algorithms. Algorithm rankings were compared using the Nemenyi tests in R mlr3benchmark package. Algorithms with similar accuracies are shown by the black bars in (A). There are no differences between dashed and solid bars. (B) shows the average rank of the algorithms by missingness levels. (C) shows the ARI distribution for cohorts with no missingness (D) shows the ARI distribution for cohorts with missingness.
(DOCX)

S14 Fig Impact of cohort type and number of classes on algorithms for BMI shape cohorts. Algorithm accuracies were compared using the Nemenyi tests in R mlr3benchmark package. Algorithms with similar accuracies are shown by the black bars in (A).
(DOCX)

S15 Fig Algorithm ranking for random glucose measurements. (A) shows the average rank of the algorithms. Algorithm rankings were compared using the Nemenyi tests in R mlr3benchmark package. Algorithms with similar accuracies are shown by the black bars in (A). There are no differences between dashed and solid bars. (B) shows the average rank of the algorithms by missingness levels. (C) shows the ARI distribution for cohorts with no missingness (D) shows the ARI distribution for cohorts with missingness.
(DOCX)

S16 Fig Impact of cohort type and number of classes on algorithms for random glucose measurements. Algorithm accuracies were compared using the Nemenyi tests in R mlr3benchmark package. Algorithms with similar accuracies are shown by the black bars in (A).
(DOCX)

S17 Fig Difference in algorithm accuracies by cohort type and number of classes for random glucose measurements. Algorithm accuracies were compared using the Nemenyi tests in R mlr3benchmark package. Algorithms with similar accuracies are shown by the black bars in (A). (A) and (B) show the difference in median ARI between magnitude and shape cohorts. ΔARI>0 indicates higher median accuracies in shape cohorts than magnitude cohorts. ΔARI is stratified by number of classes in (A). (B) shows ΔARI for combinations of centroids and distance measures used. The annotations B, M, and S refer to the algorithm combination ranking higher in both, magnitude only and shape only cohorts, respectively.
(DOCX)

S18 Fig Algorithm ranking for random glucose measurements magnitude cohorts. (A) shows the average rank of the algorithms. Algorithm rankings were compared using the Nemenyi tests in R mlr3benchmark package. Algorithms with similar accuracies are shown by the black bars in (A). There are no differences between dashed and solid bars. (B) shows the average rank of the algorithms by missingness levels. (C) shows the ARI distribution for cohorts with no missingness (D) shows the ARI distribution for cohorts with missingness.
(DOCX)

S19 Fig Impact of cohort type and number of classes on algorithms for random glucose measurements magnitude cohorts. Algorithm accuracies were compared using the Nemenyi tests in R mlr3benchmark package. Algorithms with similar accuracies are shown by the black bars in (A).
(DOCX)

S20 Fig Algorithm ranking for random glucose measurements shape cohorts. (A) shows the average rank of the algorithms. Algorithm rankings were compared using the Nemenyi tests in R mlr3benchmark package. Algorithms with similar accuracies are shown by the black bars in (A). There are no differences between dashed and solid bars. (B) shows the average rank of the algorithms by missingness levels. (C) shows the ARI distribution for cohorts with no missingness (D) shows the ARI distribution for cohorts with missingness.
(DOCX)

S21 Fig Impact of cohort type and number of classes on algorithms for random glucose measurements shape cohorts. Algorithm accuracies were compared using the Nemenyi tests in R mlr3benchmark package. Algorithms with similar accuracies are shown by the black bars in (A).
(DOCX)

S22 Fig Algorithm ranking for SBP. (A) shows the average rank of the algorithms. Algorithm rankings were compared using the Nemenyi tests in R mlr3benchmark package. Algorithms with similar accuracies are shown by the black bars in (A). There are no differences between dashed and solid bars. (B) shows the average rank of the algorithms by missingness levels. (C) shows the ARI distribution for cohorts with no missingness (D) shows the ARI distribution for cohorts with missingness.
(DOCX)

S23 Fig Impact of cohort type and number of classes on algorithms for SBP. Algorithm accuracies were compared using the Nemenyi tests in R mlr3benchmark package. Algorithms with similar accuracies are shown by the black bars in (A).
(DOCX)

S24 Fig Difference in algorithm accuracies by cohort type and number of classes for SBP. Algorithm accuracies were compared using the Nemenyi tests in R mlr3benchmark package. Algorithms with similar accuracies are shown by the black bars in (A). (A) and (B) show the difference in median ARI between magnitude and shape cohorts. ΔARI>0 indicates higher median accuracies in shape cohorts than magnitude cohorts. ΔARI is stratified by number of classes in (A). (B) shows ΔARI for combinations of centroids and distance measures used. The annotations B, M, and S refer to the algorithm combination ranking higher in both, magnitude only and shape only cohorts, respectively.
(DOCX)

S25 Fig Algorithm ranking for SBP magnitude cohorts. (A) shows the average rank of the algorithms. Algorithm rankings were compared using the Nemenyi tests in R mlr3benchmark package. Algorithms with similar accuracies are shown by the black bars in (A). There are no differences between dashed and solid bars. (B) shows the average rank of the algorithms by missingness levels. (C) shows the ARI distribution for cohorts with no missingness (D) shows the ARI distribution for cohorts with missingness.
(DOCX)

S26 Fig Impact of cohort type and number of classes on algorithms for SBP magnitude cohorts. Algorithm accuracies were compared using the Nemenyi tests in R mlr3benchmark package. Algorithms with similar accuracies are shown by the black bars in (A).
(DOCX)

S27 Fig Algorithm ranking for SBP shape cohorts. (A) shows the average rank of the algorithms. Algorithm rankings were compared using the Nemenyi tests in R mlr3benchmark package. Algorithms with similar accuracies are shown by the black bars in (A). There are no differences between dashed and solid bars. (B) shows the average rank of the algorithms by missingness levels. (C) shows the ARI distribution for cohorts with no missingness (D) shows the ARI distribution for cohorts with missingness.
(DOCX)

S28 Fig Impact of cohort type and number of classes on algorithms for SBP shape cohorts. Algorithm accuracies were compared using the Nemenyi tests in R mlr3benchmark package. Algorithms with similar accuracies are shown by the black bars in (A)
(DOCX)
