# Supplementary material for: Unsupervised clustering of longitudinal clinical measurements in electronic health records
Source: PLOS Digit Health. 2024 Oct 15;3(10):e0000628. doi: 10.1371/journal.pdig.0000628 (PMC11478862; doi:10.1371/journal.pdig.0000628)
Supplement: S1 Text — (DOCX) [file pdig.0000628.s001.docx]

# S1 Text. Supplementary Methods

The thirty algorithms in this paper are combinations of centroid types and distance measures. All combinations were applied using dtwclust package(1). More information about these methods is shown below and in S2 Table.

## Distance Measures

### Euclidean distance

Euclidean distance is the simplest distance measure for comparison of trajectories. The distance between the corresponding points of two trajectories is calculated and summed for the entire length of the trajectories(1).

### Dynamic time warping (DTW)

Dynamic time warping algorithm matches similar points on the trajectories by allowing one-to-n points mappings. The first and the last points of the two trajectories are required to match while the timepoints between these points have the flexibility to find their closest matches along the two trajectories. The distance between the first, last and closely matched timepoints is then summed to return the DTW cost. Refer to Berndt and Clifford (1994)(2) for a detailed explanation of the algorithm.

DTW is computationally expensive which limits its scalability to large datasets. Modified versions of DTW have been proposed to address this issue of scalability. These methods include specifying global constraints and lower bounds. The lower bound modifications investigated in this paper are described below.

### Lower bound- Keogh (LB-Keogh)

The Keogh lower bound method is one of the most robust lower bounding methods(3). The lower bounds and upper bounds of a trajectory are calculated. These bounds form an envelope around one of the trajectories which is used as reference. Distance is calculated between the points of the other trajectory outside the envelope and the bound of the envelope. The envelopes were calculated using Sakoe-Chiba windows of sizes varying between 2 and 15.

### Lower bound- Improved (LB-Improved)

The improved lower bounding method is faster than Keogh lower bound method. The Keogh method only calculates the envelope of a reference trajectory. This method calculates the envelops of both trajectories(4).

### DTW- Lower bound (DTW-LB)

This method uses LB-Improved for an initial estimate of the distance matrix followed by DTW between the nearest neighbors of the timeseries(1).

### Soft-Dynamic time warping (Soft-DTW)

While DTW returns the cost of optimal alignment between timeseries, soft-DTW returns the soft minimum of all possible alignments between the timeseries. Its use as a loss function has been shown to improve classification accuracies compared to other approaches(5).

### Shape based distance (SBD)

This distance measure was proposed as part of k-Shape clustering algorithm. It uses coefficient-normalized cross-correlation between the timeseries and has been shown to be faster than DTW. Please refer to Paparrizos and Gravano (2015) for more details about the algorithm(6).

### Global Alignment Kernels (GAK)

Global alignment kernels are another alternative proposed to DTW. More details can be found in the paper(7).

## References

1. Sardá-Espinosa A. Time-series clustering in R Using the dtwclust package. R Journal. 2019 Jun 1;11(1).

2. Berndt D, Clifford J. Using Dynamic Time Warping to Find Patterns in Time Series. 1994;

3. Keogh E, Ratanamahatana A. Exact indexing of dynamic time warping. Knowl Inf Syst [Internet]. 2004 [cited 2023 Jul 4];7:358–86. Available from: http://www.cs.ucr.edu/

4. Lemire D. Faster retrieval with a two-pass dynamic-time-warping lower bound. Pattern Recognit. 2009 Sep 1;42(9):2169–80.

5. Cuturi M, Blondel M. Soft-DTW: a Differentiable Loss Function for Time-Series. 2017;

6. Paparrizos J, Gravano L. k-Shape: Efficient and Accurate Clustering of Time Series.

7. Cuturi M. Fast Global Alignment Kernels. 2010;
