# Supplementary material for: Unsupervised clustering of longitudinal clinical measurements in electronic health records
Source: PLOS Digit Health. 2024 Oct 15;3(10):e0000628. doi: 10.1371/journal.pdig.0000628 (PMC11478862; doi:10.1371/journal.pdig.0000628)
Supplement: S1 Table — (DOCX) [file pdig.0000628.s002.docx]

## S1 Table. Metabolic Syndrome Criteria

| **Risk Factor** | **Measurement** | **Cut points** |
| --- | --- | --- |
| Obesity | Body mass index | >95^th^ percentile stratified by age and sex^a^ |
| Elevated blood pressure | Systolic blood pressure or  Diastolic Blood pressure | >95^th^ percentile stratified by age and sex^b^ |
| Dyslipidemia | HDL-C | <40 mg/dL (10^th^ percentile^b^) |
|  | Triglycerides  2-9 years old  10-18 years old | >100 mg/dL (5^th^ percentile^b^)  >130 mg/dL (95^th^ percentile^b^) |
| Glycemia | Fasting plasma glucose or  Random glucose  Hemoglobin A1c | >125 mg/dL  >200 mg/dL  >6.5% |

**^a^** Percentiles were retrieved from Center for Disease Control and Prevention Growth Charts

**^b^** Percentiles were retrieved from Expert Panel on Integrated Guidelines for Cardiovascular Health and Risk Reduction in Children and Adolescents (﻿EXPERT PANEL ON INTEGRATED GUIDELINES FOR CARDIOVASCULAR HEALTH AND RISK REDUCTION IN CHILDREN AND ADOLESCENTS. Expert Panel on Integrated Guidelines for Cardiovascular Health and Risk Reduction in Children and Adolescents: Summary Report. Pediatrics. 2011;128(Suppl 5):S213. doi:10.1542/PEDS.2009-2107C)
