# Supplementary material for: Unsupervised clustering of longitudinal clinical measurements in electronic health records
Source: PLOS Digit Health. 2024 Oct 15;3(10):e0000628. doi: 10.1371/journal.pdig.0000628 (PMC11478862; doi:10.1371/journal.pdig.0000628)
Supplement: S2 Table — (DOCX) [file pdig.0000628.s003.docx]

## S2 Table. Algorithms applied to simulated datasets

| **Clustering Type** | **Centroid** | **Distance Measure** |
| --- | --- | --- |
| Partitional | PAM(1)  ***Def:*** Time-series with minimum sum of distances to the other series in the group. | Euclidean(1)  DTW(2)  LB (Keogh)(3)  LB (Improved)(4)  DTW-LB(1)  Soft-DTW(5)  SBD(6)  GAK(7) |
| Fuzzy | Fuzzy C-means(8)  ***Def:*** Minimizes distance under constraints  Fuzzy C-medoids(9,10)  ***Def:*** Minimizes distance under constraints |  |
| Partitional | DBA(11)  ***Def:*** Average of points grouped according to DTW alignments. | DTW(2)  LB (Keogh)(3)  LB (Improved)(4) |
| Partitional | Soft-DTW(5)  ***Def:*** Numerical optimization using the derivative of soft-DTW. | Soft-DTW(5) |
| Partitional | Shape extraction(6)  ***Def:*** Normalized eigenvector of a matrix created with SBD-aligned series. | SBD(6) |

## References

1. Sardá-Espinosa A. Time-series clustering in R Using the dtwclust package. R Journal. 2019 Jun 1;11(1).

2. Berndt D, Clifford J. Using Dynamic Time Warping to Find Patterns in Time Series. 1994;

3. Keogh E, Ratanamahatana A. Exact indexing of dynamic time warping. Knowl Inf Syst [Internet]. 2004 [cited 2023 Jul 4];7:358–86. Available from: http://www.cs.ucr.edu/

4. Lemire D. Faster retrieval with a two-pass dynamic-time-warping lower bound. Pattern Recognit. 2009 Sep 1;42(9):2169–80.

5. Cuturi M, Blondel M. Soft-DTW: a Differentiable Loss Function for Time-Series. 2017;

6. Paparrizos J, Gravano L. k-Shape: Efficient and Accurate Clustering of Time Series.

7. Cuturi M. Fast Global Alignment Kernels. 2010;

8. Bezdek JC. Pattern Recognition with Fuzzy Objective Function Algorithms. Pattern Recognition with Fuzzy Objective Function Algorithms. 1981;

9. Izakian H, Pedrycz W, Jamal I. Fuzzy clustering of time series data using dynamic time warping distance. Eng Appl Artif Intell. 2015 Mar 1;39:235–44.

10. Krishnapuram R, Joshi A, Nasraoui O, Yi L. Low-complexity fuzzy relational clustering algorithms for Web mining. IEEE Transactions on Fuzzy Systems. 2001 Aug;9(4):595–607.

11. Petitjean FO, Ketterlin A, Ganc -Arski P. A global averaging method for dynamic time warping, with applications to clustering. Pattern Recognit [Internet]. 2010 [cited 2019 Sep 5];44:678–93. Available from: www.elsevier.com/locate/pr
