# Supplementary material for: Unsupervised clustering of longitudinal clinical measurements in electronic health records
Source: PLOS Digit Health. 2024 Oct 15;3(10):e0000628. doi: 10.1371/journal.pdig.0000628 (PMC11478862; doi:10.1371/journal.pdig.0000628)
Supplement: S3 Table — (DOCX) [file pdig.0000628.s004.docx]

## S3 Table. Descriptive statistics by MetS status

|  | **Controls** | **Cases** | **p** |
| --- | --- | --- | --- |
| N | 41,952 | 1,474 |  |
| Gender, Male N (%) | 23460 (55.9) | 735 (49.9) | <0.001 |
| Race, N (%) |  |  | 0.008 |
| American Indian or Alaska Native | 37 (0.1) | 1 (0.1) |  |
| Asians | 516 (1.2) | 17 (1.2) |  |
| Black race | 6347 (15.1) | 217 (14.7) |  |
| Caucasian | 31026 (74.0) | 1055 (71.6) |  |
| Multiracial | 2069 (4.9) | 103 (7.0) |  |
| Unknown | 1957 (4.7) | 81 (5.5) |  |
| First age, years (mean (SD)) | 4.99 (3.61) | 4.96 (3.11) | 0.808 |
| Last age, years (mean (SD)) | 13.50 (3.74) | 12.13 (2.63) | <0.001 |
| Follow-up, years (mean (SD)) | 8.52 (3.55) | 7.17 (2.88) | <0.001 |
