# Supplementary material for: Unsupervised clustering of longitudinal clinical measurements in electronic health records
Source: PLOS Digit Health. 2024 Oct 15;3(10):e0000628. doi: 10.1371/journal.pdig.0000628 (PMC11478862; doi:10.1371/journal.pdig.0000628)
Supplement: S4 Table — (DOCX) [file pdig.0000628.s005.docx]

## S4 Table. Descriptive statistics of clusters found in MetS cohort

|  | **C1** | **C2** | **C3** | **C4** | **C5** | **p** |
| --- | --- | --- | --- | --- | --- | --- |
| N | 7,558 | 7,619 | 10,952 | 10,632 | 6,665 |  |
| Gender, Male N (%) | 4119 (54.5) | 4107 (53.9) | 5479 (50.0) | 6714 (63.1) | 3776 (56.7) | <0.001 |
| Race, N (%) | | | | | | <0.001 |
| American Indian or Alaska Native | 4 (0.1) | 4 (0.1) | 11 (0.1) | 9 (0.1) | 10 (0.2) |  |
| Asians | 117 (1.5) | 76 (1.0) | 99 (0.9) | 139 (1.3) | 102 (1.5) |  |
| Black race | 1241 (16.4) | 1124 (14.8) | 2082 (19.0) | 1263 (11.9) | 854 (12.8) |  |
| Caucasian | 5519 (73.0) | 5692 (74.7) | 7517 (68.6) | 8308 (78.1) | 5045 (75.7) |  |
| Multiracial | 369 (4.9) | 372 (4.9) | 655 (6.0) | 440 (4.1) | 336 (5.0) |  |
| Unknown | 308 (4.1) | 351 (4.6) | 588 (5.4) | 473 (4.4) | 318 (4.8) |  |
| First age, years (mean (SD)) | 3.48 (2.61) | 5.43 (3.69) | 6.29 (3.90) | 3.98 (2.86) | 5.64 (3.91) | <0.001 |
| Last age, years (mean (SD)) | 12.97 (3.66) | 13.57 (3.72) | 13.52 (3.76) | 13.84 (3.38) | 13.17 (4.11) | <0.001 |
| Follow-up, years (mean (SD)) | 9.49 (3.51) | 8.14 (3.42) | 7.23 (3.19) | 9.86 (3.42) | 7.53 (3.34) | <0.001 |
| MetS, N (%) | 260 (3.4) | 246 (3.2) | 685 (6.3) | 193 (1.8) | 90 (1.4) | <0.001 |
