# Supplementary material for: Unsupervised clustering of longitudinal clinical measurements in electronic health records
Source: PLOS Digit Health. 2024 Oct 15;3(10):e0000628. doi: 10.1371/journal.pdig.0000628 (PMC11478862; doi:10.1371/journal.pdig.0000628)
Supplement: S5 Table — (DOCX) [file pdig.0000628.s006.docx]

## S5 Table. Descriptive statistics of clusters in MetS cases only

|  | **C1** | **C2** | **C3** | **C4** | **C5** | **p** |
| --- | --- | --- | --- | --- | --- | --- |
| N | 260 | 246 | 685 | 193 | 90 |  |
| Gender, Male N (%) | 117  (45.0) | 128 (52.0) | 316 (46.1) | 117 (60.6) | 57 (63.3) | <0.001 |
| Race, N (%) |  |  |  |  |  | 0.059 |
| American Indian or Alaska Native | 0 (0.0) | 0 (0.0) | 0 (0.0) | 1 (0.5) | 0 (0.0) |  |
| Asians | 7 (2.7) | 2 (0.8) | 4 (0.6) | 2 (1.0) | 2 (2.2) |  |
| Black race | 40 (15.4) | 34 (13.8) | 114 (16.6) | 23 (11.9) | 6 (6.7) |  |
| Caucasian | 185 (71.2) | 185 (75.2) | 468 (68.3) | 147 (76.2) | 70 (77.8) |  |
| Multiracial | 15 (5.8) | 16 (6.5) | 56 (8.2) | 11 (5.7) | 5 (5.6) |  |
| Unknown | 13 (5.0) | 9 (3.7) | 43 (6.3) | 9 (4.7) | 7 (7.8) |  |
| First age, years (mean (SD)) | 3.47 (2.43) | 5.20 (3.24) | 5.53 (3.15) | 4.11 (2.62) | 6.18 (3.25) | <0.001 |
| Last age, years (mean (SD)) | 11.72 (2.61) | 12.36 (2.64) | 11.99 (2.72) | 12.58 (2.38) | 12.82 (2.31) | <0.001 |
| Follow-up, years (mean (SD)) | 8.25 (2.74) | 7.16 (2.87) | 6.46 (2.62) | 8.47 (2.97) | 6.64 (3.07) | <0.001 |
