# Supplementary figures and images for: Unsupervised clustering of longitudinal clinical measurements in electronic health records
Source: PLOS Digit Health. 2024 Oct 15;3(10):e0000628. doi: 10.1371/journal.pdig.0000628 (PMC11478862; doi:10.1371/journal.pdig.0000628)

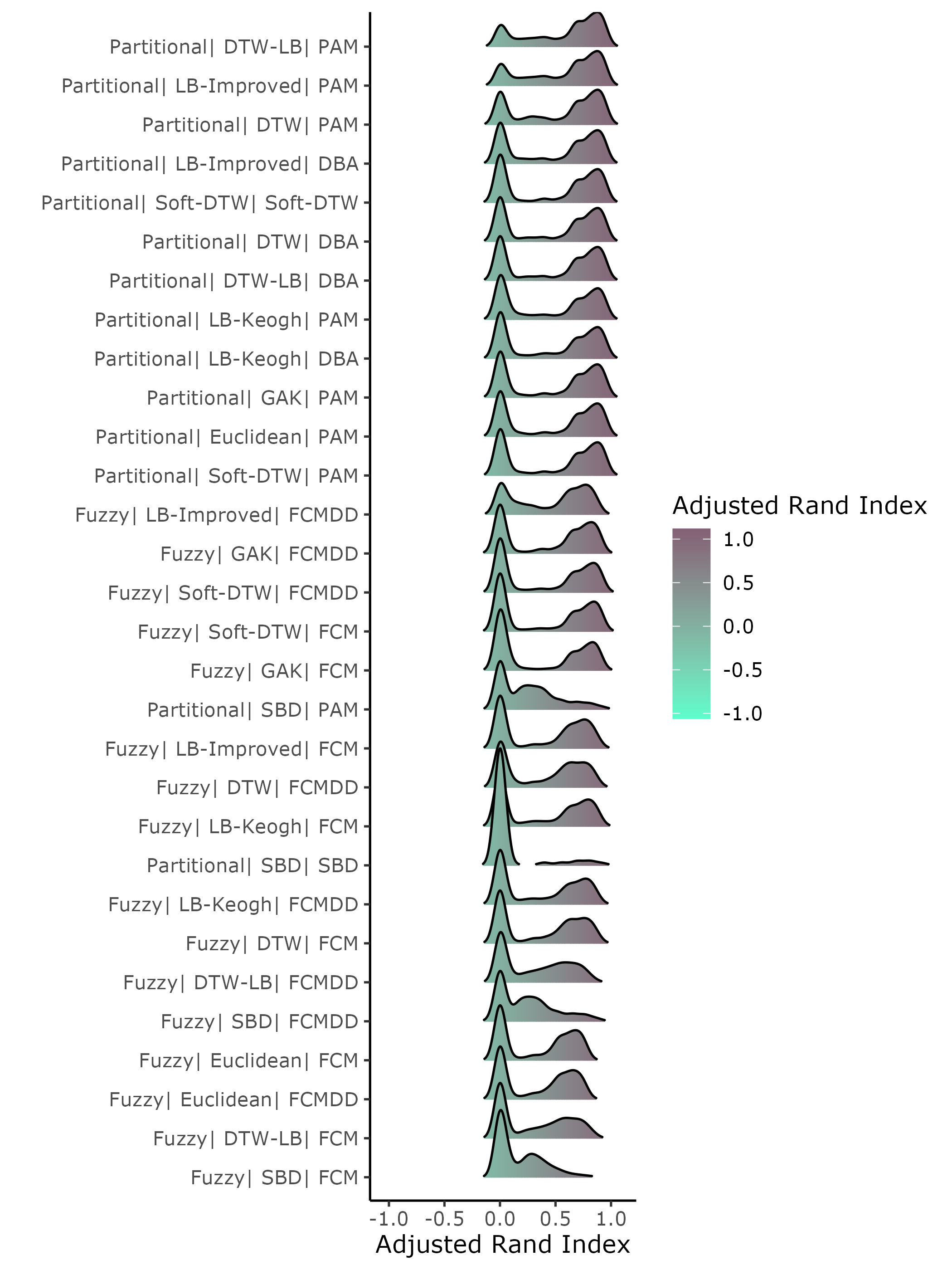


## S1 Fig. Adjusted Rand Index Distributions for algorithms for all cohorts

Supplement: S1 Fig — (DOCX) [file pdig.0000628.s007.docx]

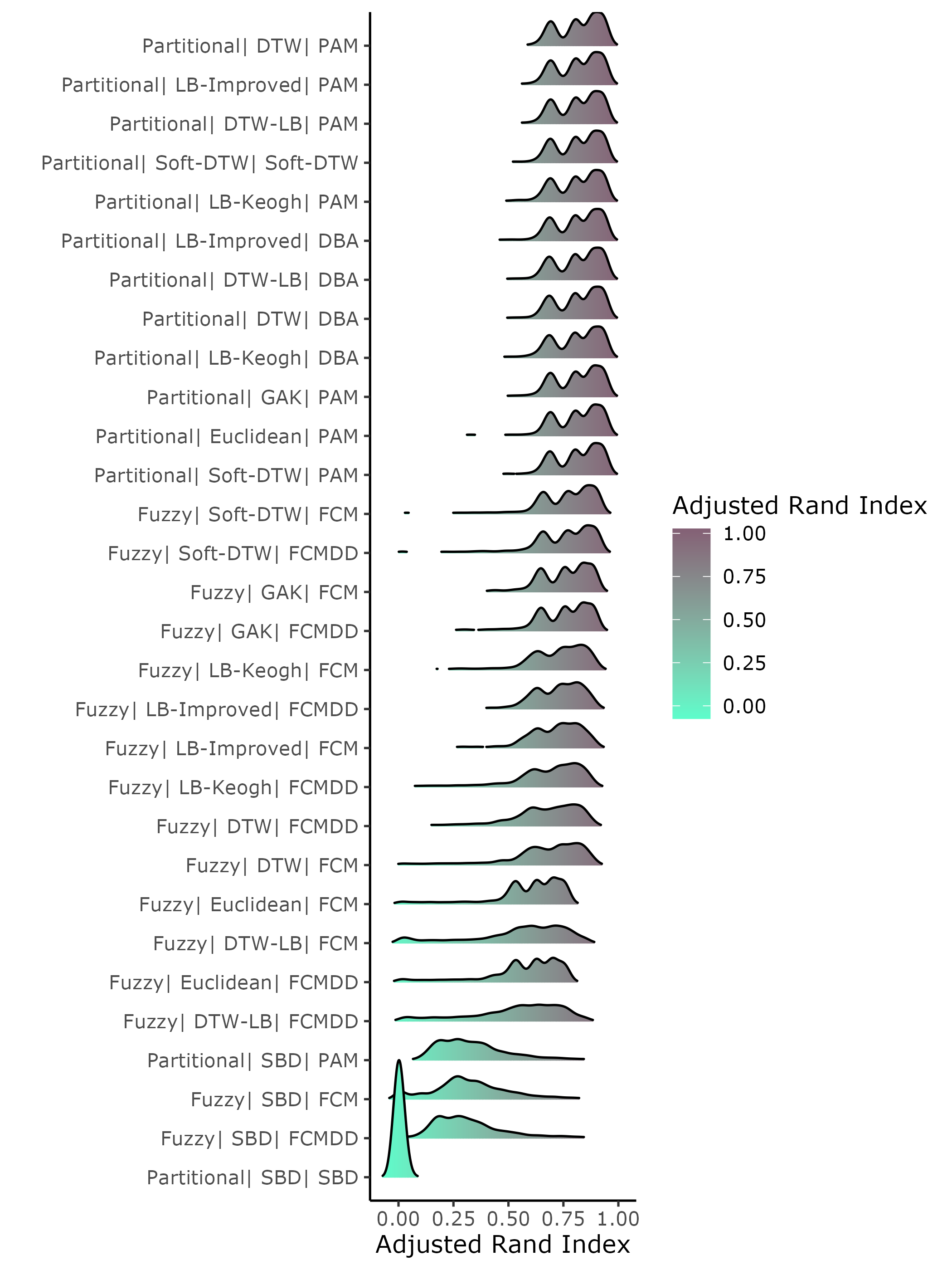


## S4 Fig. Adjusted Rand Index Distributions for algorithms for all magnitude cohorts

Supplement: S4 Fig — (DOCX) [file pdig.0000628.s010.docx]

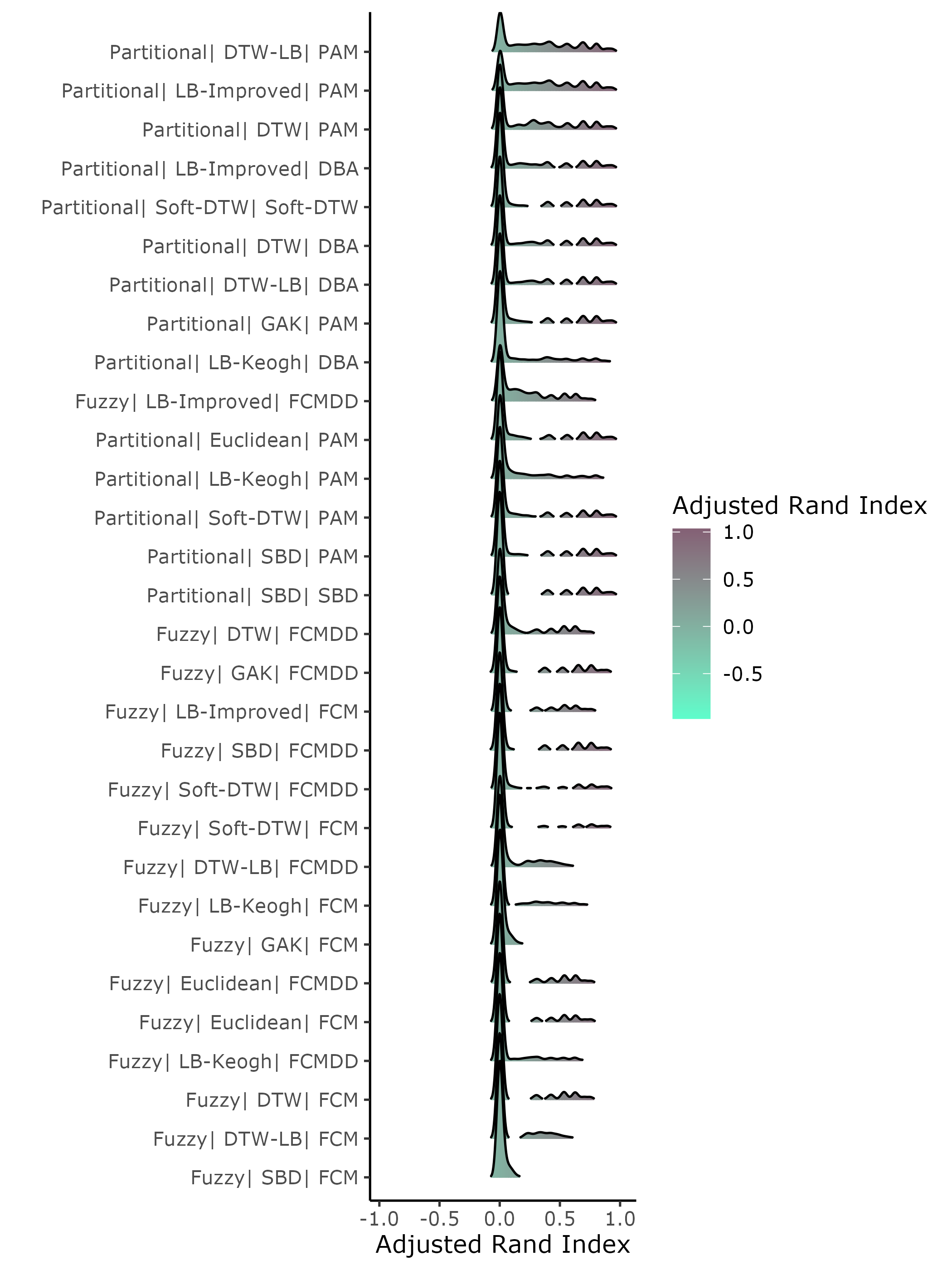


## S7 Fig. Adjusted Rand Index Distributions for algorithms for all shape cohorts

Supplement: S7 Fig — (DOCX) [file pdig.0000628.s013.docx]
