# Supplementary material for: Unsupervised clustering of longitudinal clinical measurements in electronic health records
Source: PLOS Digit Health. 2024 Oct 15;3(10):e0000628. doi: 10.1371/journal.pdig.0000628 (PMC11478862; doi:10.1371/journal.pdig.0000628)
Supplement: S22 Fig — (A) shows the average rank of the algorithms. Algorithm rankings were compared using the Nemenyi tests in R mlr3benchmark package. Algorithms with similar accuracies are shown by the black bars in (A). There are no differences between dashed and solid bars. (B) shows the average rank of the algorithms by missingness levels. (C) shows the ARI distribution for cohorts with no missingness (D) shows the ARI distribution for cohorts with missingness. (DOCX) [file pdig.0000628.s028.docx]

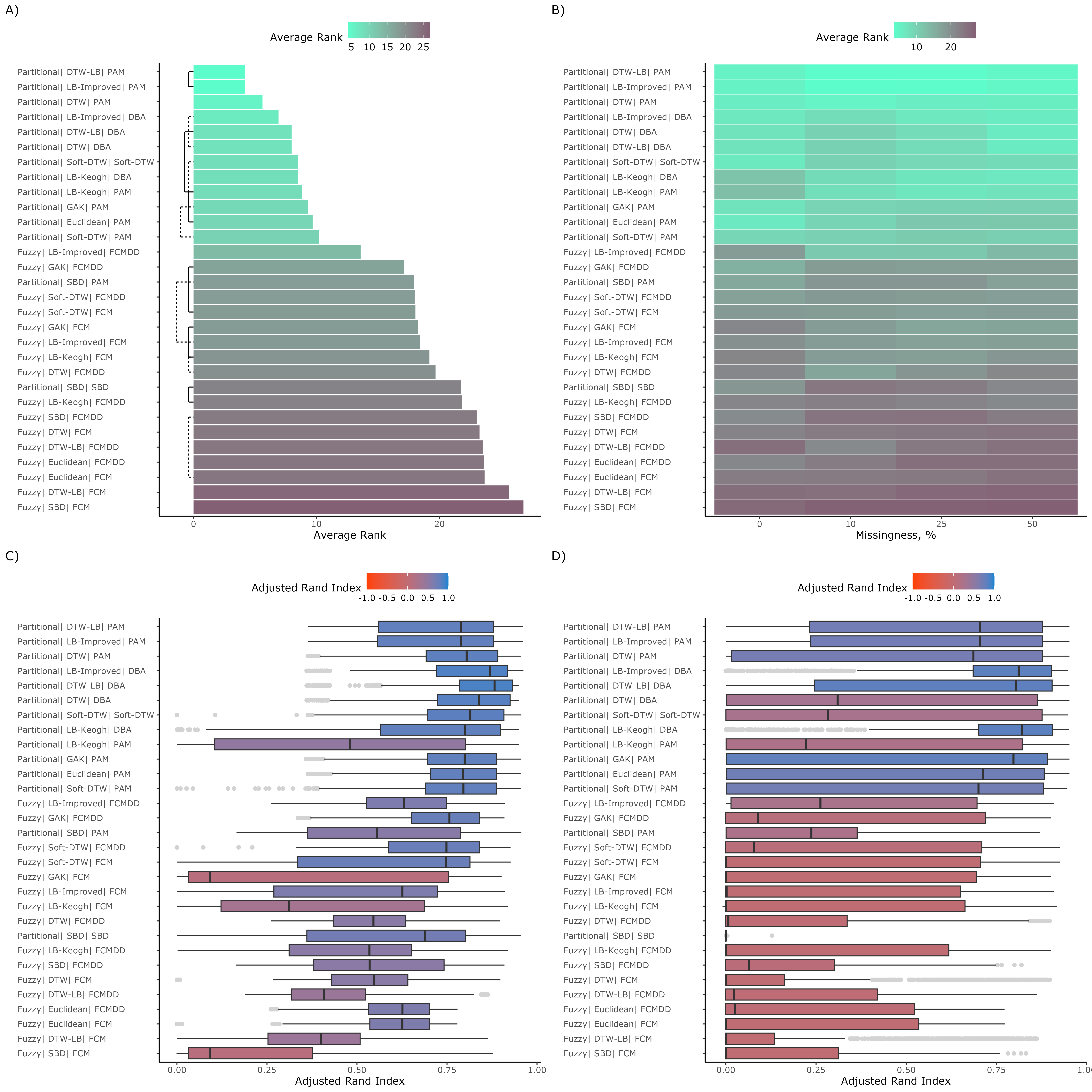


## S22 Fig. Algorithm ranking for SBP

(A) shows the average rank of the algorithms. Algorithm rankings were compared using the Nemenyi tests in R mlr3benchmark package. Algorithms with similar accuracies are shown by the black bars in (A). There are no differences between dashed and solid bars. (B) shows the average rank of the algorithms by missingness levels. (C) shows the ARI distribution for cohorts with no missingness (D) shows the ARI distribution for cohorts with missingness.
