# Supplementary material for: Unsupervised clustering of longitudinal clinical measurements in electronic health records
Source: PLOS Digit Health. 2024 Oct 15;3(10):e0000628. doi: 10.1371/journal.pdig.0000628 (PMC11478862; doi:10.1371/journal.pdig.0000628)
Supplement: S24 Fig — Algorithm accuracies were compared using the Nemenyi tests in R mlr3benchmark package. Algorithms with similar accuracies are shown by the black bars in (A). (A) and (B) show the difference in median ARI between magnitude and shape cohorts. ΔARI>0 indicates higher median accuracies in shape cohorts than magnitude cohorts. ΔARI is stratified by number of classes in (A). (B) shows ΔARI for combinations of centroids and distance measures used. The annotations B, M, and S refer to the algorithm combination ranking higher in both, magnitude only and shape only cohorts, respectively. (DOCX) [file pdig.0000628.s030.docx]

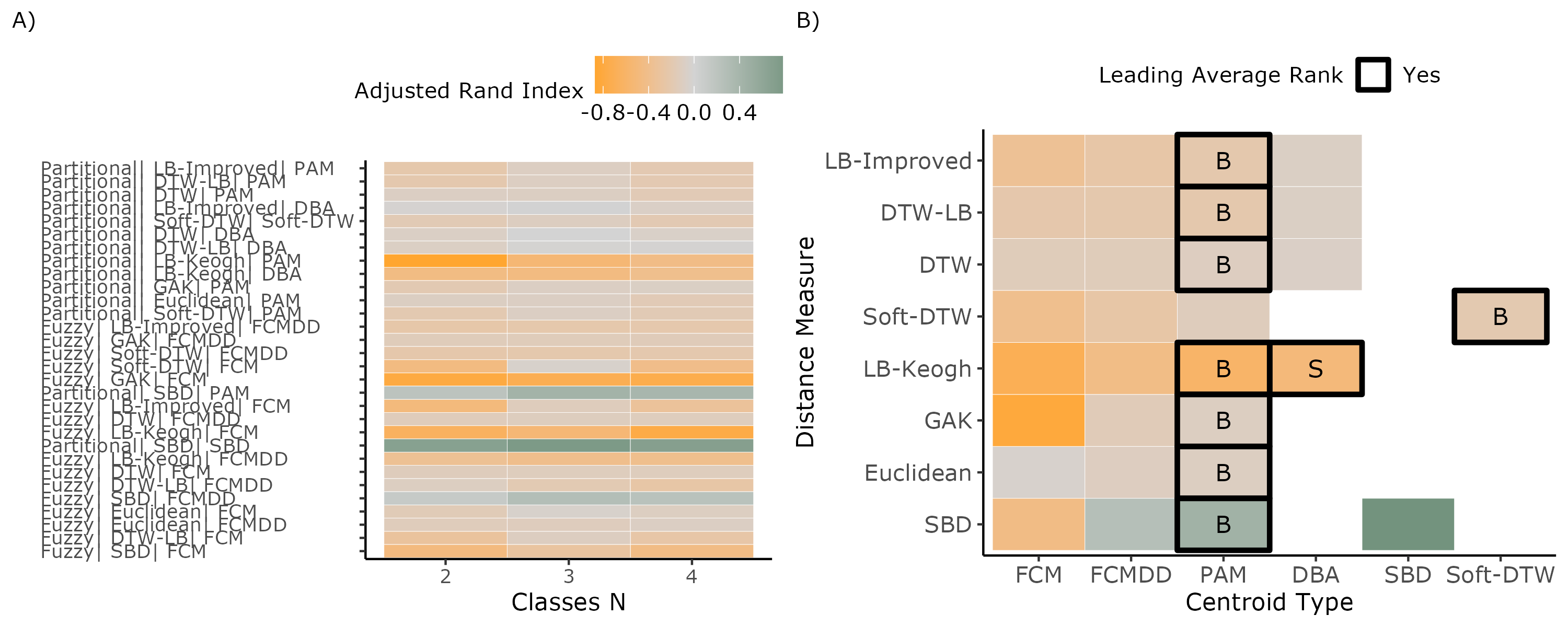


## S24 Fig. Difference in algorithm accuracies by cohort type and number of classes for SBP

Algorithm accuracies were compared using the Nemenyi tests in R mlr3benchmark package. Algorithms with similar accuracies are shown by the black bars in (A). (A) and (B) show the difference in median ARI between magnitude and shape cohorts. ΔARI>0 indicates higher median accuracies in shape cohorts than magnitude cohorts. ΔARI is stratified by number of classes in (A). (B) shows ΔARI for combinations of centroids and distance measures used. The annotations B, M, and S refer to the algorithm combination ranking higher in both, magnitude only and shape only cohorts, respectively.
