# Supplementary material for: Unsupervised clustering of longitudinal clinical measurements in electronic health records
Source: PLOS Digit Health. 2024 Oct 15;3(10):e0000628. doi: 10.1371/journal.pdig.0000628 (PMC11478862; doi:10.1371/journal.pdig.0000628)
Supplement: S26 Fig — Algorithm accuracies were compared using the Nemenyi tests in R mlr3benchmark package. Algorithms with similar accuracies are shown by the black bars in (A). (DOCX) [file pdig.0000628.s032.docx]

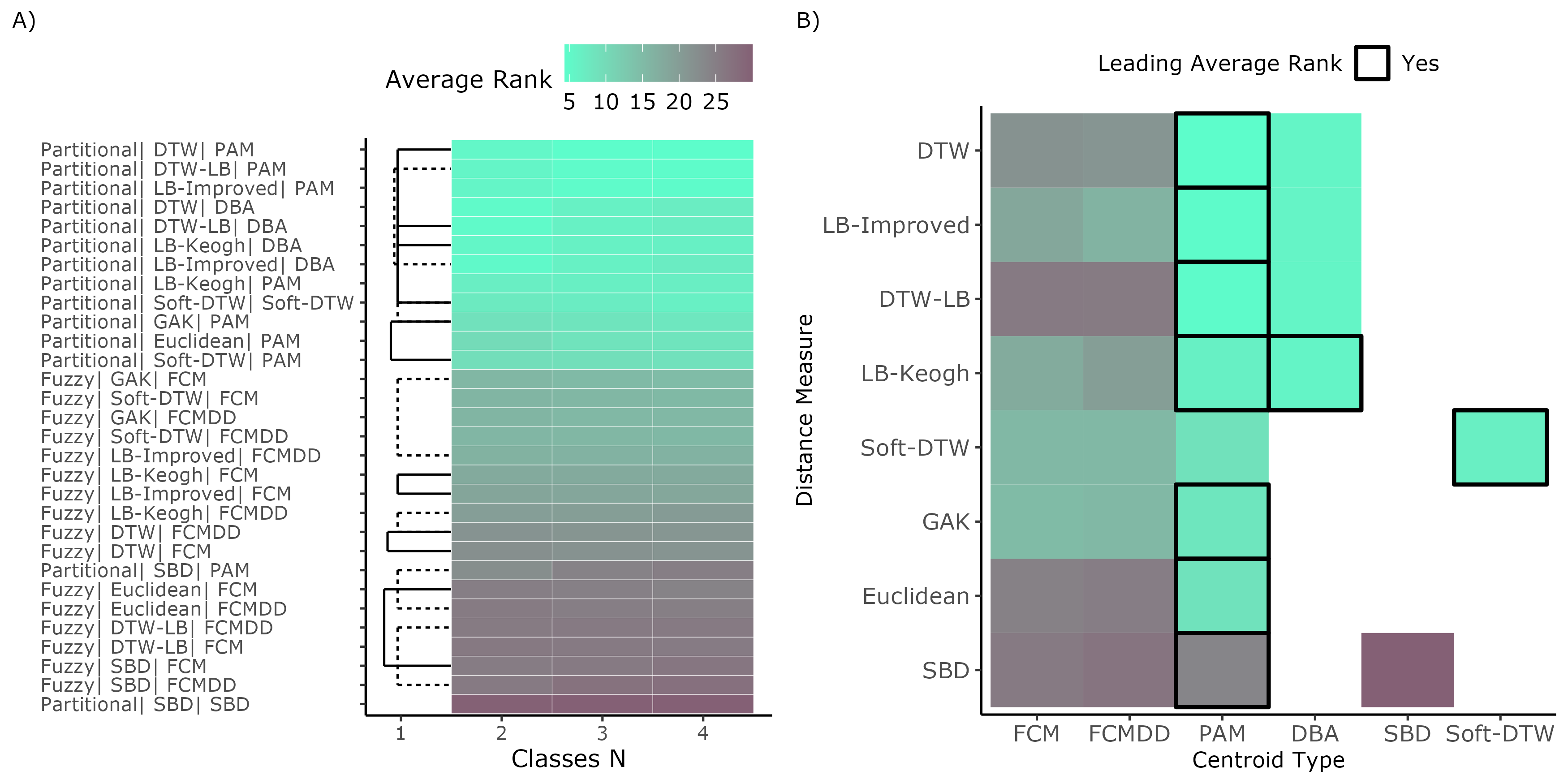


## S26 Fig. Impact of cohort type and number of classes on algorithms for SBP magnitude cohorts

Algorithm accuracies were compared using the Nemenyi tests in R mlr3benchmark package. Algorithms with similar accuracies are shown by the black bars in (A).
